# An RxLR Effector from *Phytophthora infestans* Prevents Re-localisation of Two Plant NAC Transcription Factors from the Endoplasmic Reticulum to the Nucleus

**DOI:** 10.1371/journal.ppat.1003670

**Published:** 2013-10-10

**Authors:** Hazel McLellan, Petra C. Boevink, Miles R. Armstrong, Leighton Pritchard, Sonia Gomez, Juan Morales, Stephen C. Whisson, Jim L. Beynon, Paul R. J. Birch

**Affiliations:** 1 The Division of Plant Sciences, College of Life Science, University of Dundee at the James Hutton Institute, Invergowrie, Dundee, United Kingdom; 2 Dundee Effector Consortium, James Hutton Institute, Invergowrie, Dundee, United Kingdom; 3 Cell and Molecular Sciences, James Hutton Institute, Invergowrie, Dundee, United Kingdom; 4 Information and Computational Sciences, JHI, Invergowrie, Dundee, United Kingdom; 5 Departamento de Ciencias Agronómicas, Universidad Nacional de Colombia, Sede Medellin, Medellin, Colombia; 6 Life Sciences and Systems Biology, University of Warwick, Coventry, United Kingdom; Oregon State University, United States of America

## Abstract

The potato late blight pathogen *Phytophthora infestans* secretes an array of effector proteins thought to act in its hosts by disarming defences and promoting pathogen colonisation. However, little is known about the host targets of these effectors and how they are manipulated by the pathogen. This work describes the identification of two putative membrane-associated NAC transcription factors (TF) as the host targets of the RxLR effector PITG_03192 (Pi03192). The effector interacts with NAC Targeted by *Phytophthora* (NTP) 1 and NTP2 at the endoplasmic reticulum (ER) membrane, where these proteins are localised. Transcripts of *NTP1* and *NTP2* rapidly accumulate following treatment with culture filtrate (CF) from *in vitro* grown *P. infestans*, which acts as a mixture of *Phytophthora* PAMPs and elicitors, but significantly decrease during *P. infestans* infection, indicating that pathogen activity may prevent their up-regulation. Silencing of *NTP1* or *NTP2* in the model host plant *Nicotiana benthamiana* increases susceptibility to *P. infestans*, whereas silencing of *Pi03192* in *P. infestans* reduces pathogenicity. Transient expression of Pi03192 *in planta* restores pathogenicity of the *Pi03192-*silenced line. Moreover, colonisation by the *Pi03192-*silenced line is significantly enhanced on *N. benthamiana* plants in which either *NTP1* or *NTP2* have been silenced. StNTP1 and StNTP2 proteins are released from the ER membrane following treatment with *P. infestans* CF and accumulate in the nucleus, after which they are rapidly turned over by the 26S proteasome. In contrast, treatment with the defined PAMP flg22 fails to up-regulate *NTP1* and *NTP2*, or promote re-localisation of their protein products to the nucleus, indicating that these events follow perception of a component of CF that appears to be independent of the FLS2/flg22 pathway. Importantly, Pi03192 prevents CF-triggered re-localisation of StNTP1 and StNTP2 from the ER into the nucleus, revealing a novel effector mode-of-action to promote disease progression.

## Author Summary

The plant immune system is activated following the perception of exposed, essential and invariant microbial molecules that are recognised as non-self. A major component of plant immunity is the transcriptional induction of genes involved in a wide array of defence responses. In turn, adapted pathogens deliver effector proteins that act either inside or outside plant cells to manipulate host processes, often through their direct action on plant protein targets. To date, few effectors have been shown to directly manipulate transcriptional regulators of plant defence. Moreover, little is known generally about the modes of action of effectors from filamentous (fungal and oomycete) plant pathogens. We describe an effector, called Pi03192, from the late blight pathogen *Phytophthora infestans*, which interacts with a pair of host transcription factors at the endoplasmic reticulum (ER) inside plant cells. We show that these transcription factors are released from the ER to enter the nucleus, following pathogen perception, and are important in restricting disease. Pi03192 prevents the plant transcription factors from accumulating in the host nucleus, revealing a novel means of enhancing host susceptibility.

## Introduction

Lacking an adaptive immune system, plants have evolved a two-tier surveillance system to detect and deflect pathogen incursions. The first layer is triggered by receptor-like kinase pattern recognition receptors (RLK-PRRs), which recognise conserved non-self molecules or pathogen associated molecular patterns (PAMPs) [1]–[3]. These PRRs enable plants to sense the proximity of potential pathogenic microbes and activate their defences accordingly, for example by production of reactive oxygen species, callose deposition and synthesis of antimicrobial compounds [4]. This is termed PAMP-triggered immunity (PTI) [5] and is thought to be effective in restricting the growth of the majority of would-be pathogens. However, dedicated plant pathogenic organisms have evolved the ability to secrete a range of effector molecules that suppress PTI. Some, such as bacterial type III effectors [6] and oomycete RxLR effectors [7]–[8], are translocated inside plant cells and are believed often to attenuate PTI by manipulating host target proteins; this is termed effector triggered susceptibility (ETS) [5]. In response, a second layer of resistance has evolved in plants comprising resistance (R) proteins which detect effectors, or their activity, often resulting in a localised cell death or hypersensitive response (HR). This is termed effector-triggered immunity (ETI) [5], [9]–[11]. This model for plant-pathogen interactions is summarised for plant-oomycete interactions in Hein et al. [12]. However, to date, little is known about how oomycete effectors alter plant defence or metabolism to their own ends.

Following the identification of oomycete RxLR effectors, and the demonstration that they are delivered inside plant cells [13]–[15], effort has been directed at defining the repertoire of this class of effectors in the genomes of diverse oomycete species [16]–[18]. Yet little is known about the virulence function of these proteins within the plant host. Several relatively high throughput assays have been carried out to establish possible roles for RxLR effectors in the suppression of PTI and ETI [19]–[21]. Screens of *Phytophthora sojae* RxLRs found the majority of effectors were able to suppress cell death (CD) triggered by a range of CD-inducing elicitors in transient assays in *Nicotiana benthamiana* [20]. In addition, 70% of tested *Hyaloperonospora arabidopsidis* RxLRs were found to supress PTI by enhancing the growth of *Pseudomonas* in different ecotypes of *Arabidopsis thaliana* [21]. While such studies support the hypothesis that pathogens secrete effectors to manipulate plant defence, they do not shed light on the mechanisms behind this.

Recently, large-scale matrix yeast-two-hybrid (M2H) screens were conducted with the aim of mapping plant protein-protein interaction networks and identifying the likely “hubs” or convergence points of these networks targeted for manipulation by plant pathogens [22]–[23]. Of >8000 screened Arabidopsis proteins 17 were found to interact with effectors from both *Pseudomonas syringae* and *H. arabidopsidis*, with 15 of these showing defence-associated phenotypes in plant knockout studies [23], indicating that pathogens separated by 2 billion years of evolution have potentially developed similar strategies for manipulating their host. However, given that 165 putative host targets were identified in this screen [23], the question of which interactions are relevant *in planta*, and the precise mode of action of these effectors remains open.

The little that is known about the manipulation of plant targets by oomycete effectors has mainly concentrated on those RxLRs which are recognised by plant resistance proteins. One of the best studied oomycete RxLRs, AVR3a from *Phytophthora infestans*, was found to interact with and stabilise the host ubiquitin E3 ligase CMPG1, interfering with its ability to mediate cell death in response to perception of pathogen elicitors at the host cell membrane [24]–[25]. Recently another *P. infestans* RxLR, AVR2, was found to interact with the plant phosphatase BSL1 and this interaction appears to be recognised or “guarded” by the resistance protein R2 [26]. The RxLR effector Avrblb2 from *P. infestans* prevents secretion into the plant apoplast of the plant defence-related protease C14 [27]. In addition, the avirulence protein Avr3b from *P. sojae* is an ADP-ribose/NADH pyrophosphorylase and is predicted to modulate plant immunity through this activity [28]. A role in virulence has also been proposed for the *P. infestans* effector Avrblb1 (IPIO1) through the disruption of plasma membrane/cell wall adhesions *via* its interaction with the lectin receptor kinase LecRK-I.9 [29]–[30].

Studies of candidate RxLR effectors from *H. arabidopsidis* reveal that they localise to a number of subcellular locations, potentially indicating a diversity of host targets and modes of effector activity [31]. Of 49 candidate RxLRs, 33% were localised strictly in the plant nucleus and a further 33% were nucleo-cytoplasmic. The majority of the remaining RxLRs (26%) were associated with the plant membrane trafficking network, most of which (18%) were localised to the endoplasmic reticulum [31]. The large number of *H. arabidopsidis* candidate RxLR effectors localised to the plant nucleus is perhaps unsurprising; transcriptional changes are at the heart of PTI [6], also many regulatory components of plant immunity that are active in the nucleus are trafficked there from a variety of subcellular locations [32]–[33].

In this study we demonstrate a novel virulence function for the *P. infestans* RxLR PITG_03192 (Pi03192). Yeast-2-hybrid and bimolecular fluorescence complementation (BiFC) analyses revealed two putative membrane-associated NAC (NAM/ATAF/CUC) transcription factors (TFs) as the potato host targets of Pi03192. Interactions with these NAC TFs occur at the endoplasmic reticulum (ER) membrane. Virus induced gene silencing (VIGS) of the genes encoding these two putative TFs leads to an increase in susceptibility to *P. infestans* infection, suggesting these NAC TFs play a role in plant defence. Silencing of *Pi03192* in *P. infestans* results in a reduction of virulence on potato and *N. benthamiana*. Critically, virulence of the silenced line on *N. benthamiana* can be enhanced by VIGS of either *NTP1* or *NTP2*. Both NAC targeted by *P. infestans* (*NTP*) *1* and *NTP2* transcripts accumulate following treatment with culture filtrate (CF) from *in vitro*-grown *P. infestans*, which potentially contains a mixture of *Phytophthora* PAMPs and elicitors, but not following treatment with flg22. In contrast, transcripts of both genes decrease during the early stages of *P. infestans* colonisation, suggesting that the pathogen manipulates transcription levels of these genes. On treatment with CF, but not flg22, the putative ER membrane-associated NAC TFs, NTP1 and NTP2 translocate to the nucleus, where they are rapidly turned over by the 26S proteasome. We show that Pi03192 promotes virulence by preventing the nuclear accumulation of the two NAC TFs.

## Results

### Pi03192 promotes *P. infestans* host colonisation and interacts with two potato NAC transcription factors

Candidate RXLR effector PITG_03192 (Pi03192), also named RD28 [19], was one of the first predicted from *P. infestans* expressed sequence tags [14]. In keeping with the expression profiles of most RXLR effector genes, *Pi03192* transcripts accumulate specifically during the first 3 days of *P. infestans* infection of potato [14], [19] and tomato [19].

**Figure 1:**
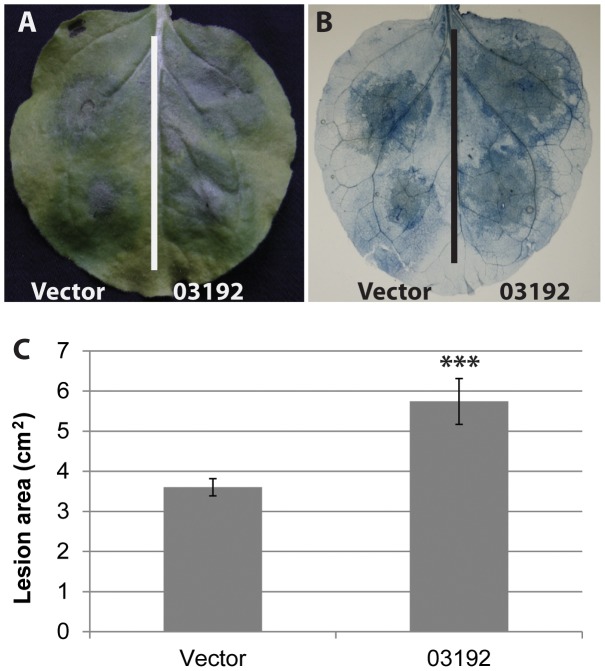
Transient over-expression of Pi03192 *in planta* increases *P. infestans* virulence. Agrobacterium was used to transiently over-express Pi03192 on one half of a leaf and empty vector on the other. Leaves were subsequently infected with *P. infestans*. (A) Shows a typical leaf with larger lesions on the half of the leaf expressing Pi03192. (B) The leaf in (A) stained with trypan blue to show increased *P. infestans* mycelial growth on the half of the leaf expressing Pi03192. (C) Graph quantifying lesion area in the presence or absence of Pi03192 expression. Error bars show standard error and asterisks indicate a highly significant difference (p<0.001, t-test) (from 3 biological replicates each comprising 17 inoculations per treatment).

*Nicotiana benthamiana* is a model for functional studies in the *Solanaceae*, and is also a host for *P. infestans*. As such it has been extensively used to investigate pathogen and host gene functions in *P. infestans*-plant interactions [19], [24], [26]–[27], [34]. To investigate whether Pi03192 acts to promote *P. infestans* colonisation the effector gene, minus signal peptide-encoding sequences, was expressed transiently in *N. benthamiana*. Figure 1 shows that, compared to expression of an empty vector control, *Agrobacterium*-mediated expression of Pi03192 inside *N. benthamiana* cells significantly enhances *P. infestans* colonisation by 9 days post-inoculation (dpi). This suggests that Pi03192, as a predicted translocated RxLR effector, acts within plant cells to promote ETS, prompting us to investigate its mode-of-action and potential host targets.

**Figure 2:**
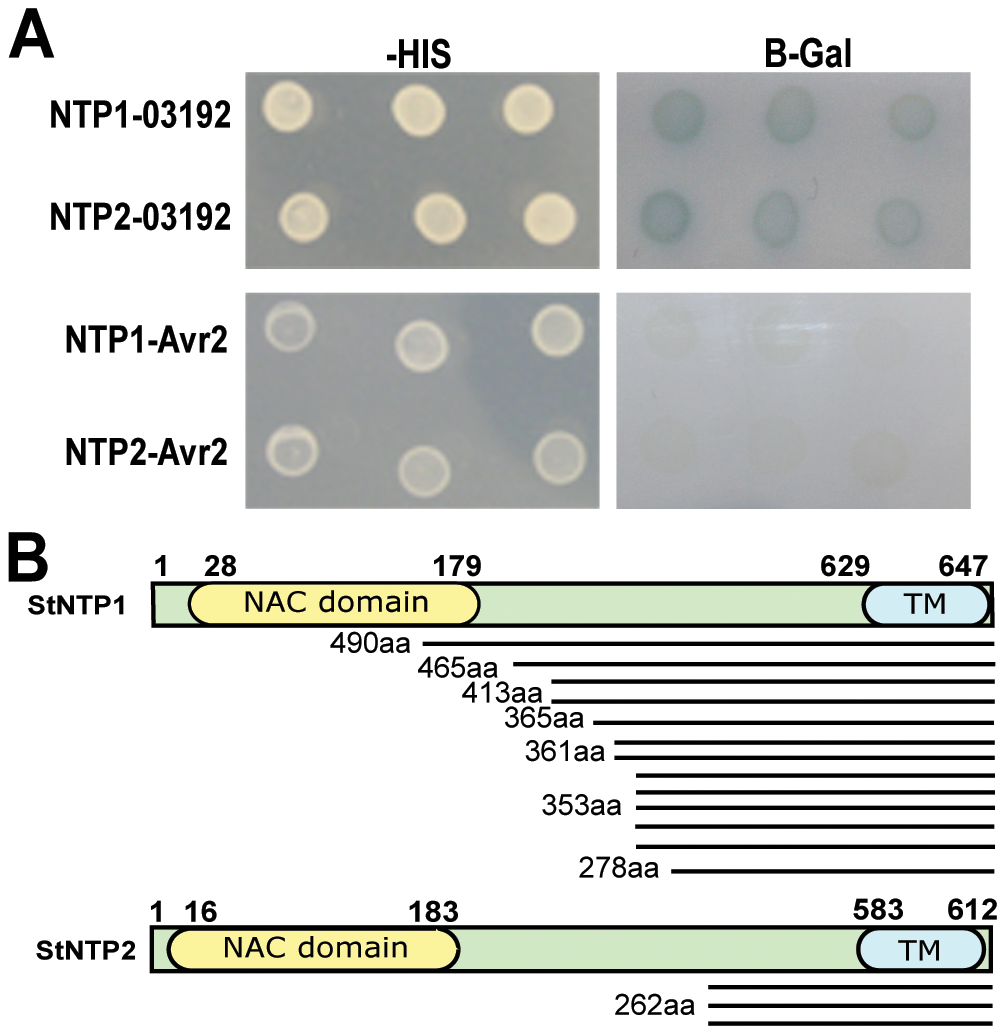
Pi03192 interacts with two putative NAC transcription factors from potato. (A) StNTP1 and StNTP2 grow on Δ histidine (-HIS) media and show β-galactosidase (B-Gal) activity when co-transformed with Pi03192 but not with control RXLR effector PiAvr2. (B) Schematic representations of StNTP1 and StNTP2 showing NAM DNA-binding domains (NAC domain) and TM domains and the number, length in amino acids (aa) and position of the interacting fragments identified in Y2H.

To identify candidate host targets of *P. infestans* RxLR effectors a yeast-2-hybrid (Y2H) library composed of cDNA from potato plants infected with *P. infestans* [24] was screened with the candidate RxLR effector Pi03192. After screening 8×10^6^ yeast transformants, 16 clones expressing interacting proteins were identified. Sequences from 13 of 16 clones were found to encode the C-terminal region of a predicted membrane-associated NAC transcription factor (TF), and the remaining 3 clones were found to encode the C terminus of a distinct predicted membrane-associated NAC TF (Figure 2A and B). These NAC TF fragments interacted with Pi03192 but did not interact with another verified RxLR effector, PiAVR2 (Figure 2A), which was used as a control. BLAST searches and alignments of ESTs from related solanaceous species tomato, potato and petunia were used to design primers to amplify the full-length potato NAC TF genes. Both genes were found to encode proteins containing a predicted N-terminal NAC DNA binding (NAM) domain and a predicted transmembrane (TM) domain at the C terminus of the protein (Figure 2B). These genes were thus tentatively designated *Solanum tuberosum NAC Targeted by Phytophthora* (*StNTP*) *1* (13/16 clones) and *StNTP2* (3/16 clones).

To further explore the relationship of StNTP1 and StNTP2 with NAC TFs from other plant species a phylogenetic tree was constructed by maximum likelihood based on the 337 most similar NAM domains extracted from plant sequences contained in the NCBI RefSeq sequence database (Figure S1 and S2). Both StNTP1 and StNTP2 are placed in clades with little or no representation of cereal NAC TF sequences. In each case the potato NAC sequences form a clade with NAC TFs from other *Solanaceae*, which are paired with a similarly expanded grouping of *Arabidopsis thaliana* NACs. In *Arabidopsis thaliana* 13 genes encoding proteins with an N-terminal NAM domains and C-terminal TM domains (termed NTL1-13) have previously been reported, and classified into four phylogenetic subgroups [35]. The phylogenetic tree in Figure S1 differs from that proposed by Kim et al. [35], and incorporates additional *A. thaliana* candidate *NTLs* not included in that analysis. This improved tree places StNTP1 in a clade with AtNTL6, and StNTP2 in a clade that contains the three Arabidopsis NACs AtNTL1, AtNTL3 and AtNTL7 (Figure S1 and S2) Both clades contain sequences from a number of plant species, with or without predicted transmembrane domains at the C-terminal region. An alignment of the NAM domains of StNTP1 and StNTP2 with the 13 reported AtNTLs shows that the potato NAM domains contain the critical conserved residues required for DNA binding and for NAC TF dimerization (Figure S3).

### Pi03192, StNTP1 and StNTP2 localise to the ER membrane *in planta*

**Figure 3:**
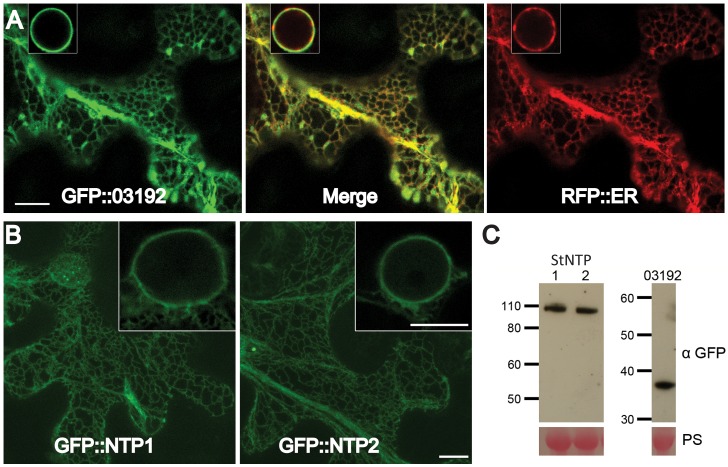
StNTP1, StNTP2 and Pi03192 are localised to the ER membrane *in planta*. (A) GFP-Pi03192 co-localises to the ER membrane with an RFP tagged ER marker. Scale bars indicate 10 µm, and insert-images show ER around the nucleus. (B) GFP-StNTP1 and 2 localise to the ER and are found in this membrane surrounding the nucleus (insert-images). Scale bars indicate 10 µm. (C) Immunoblots of GFP-StNTP1, GFP-StNTP2 and GFP-Pi03192 showing the stability of the full length constructs when probed with a specific GFP antibody. PS is Ponceau stain.

The subcellular localisations of StNTP1, StNTP2 and Pi03192 were examined by *Agrobacterium*-mediated transient expression of each with N-terminal GFP fusions in *N. benthamiana* and imaging using confocal microscopy. Images of cells expressing each of the constructs revealed a network of fluorescence suggestive of localisation to the endoplasmic reticulum (ER) membrane *in planta* (Figure 3A and B). To confirm the ER localisation of the RxLR effector, GFP-Pi03192 was co-expressed with an RFP-ER-tagged construct and imaged by confocal microscopy. Both were observed to co-localise to the ER network as shown by the merge of the green and red channels (Figure 3A). The ER membrane localisation is consistent with the observation that both StNTPs possess predicted C-terminal TM domains. However, GFP-Pi03192 is also ER localised despite the absence of a predicted TM domain. Western blots hybridised with an antibody specific to GFP demonstrated that the GFP-StNTP1, GFP-StNTP2 and GFP-Pi03192 fusion proteins were stable, showing bands of the predicted sizes, respectively 111 kDa, 106 kDa and 39 kDa (Figure 3C).

As NAC TFs are expected to localise to the nucleus following release from the ER, N-terminal fusions of GFP to StNTP1 and StNTP2 lacking the predicted TM domains were made. Surprisingly, confocal images showed no fluorescence of GFP-StNTP1ΔTM and GFP-*St*NTP2ΔTM (Figure S4A). However, literature regarding membrane-associated transcription factors (MTFs) suggests that many are tightly regulated and rapidly turned over by the 26S proteasome [36]–[38]. When leaves expressing the GFP-StNTPΔTM constructs were treated with the proteasome inhibitor MG132 fluorescence was clearly observed, as anticipated, exclusively in the nucleus (Figure S4A). Immunoblots to indicate fusion protein stability could only detect GFP-StNTPΔTMs in the presence of the proteasome inhibitor (Figure S4B). This suggests that activity of these StNTPs is regulated *in planta*, at least in part, by rapid protein turnover.

**Figure 4:**
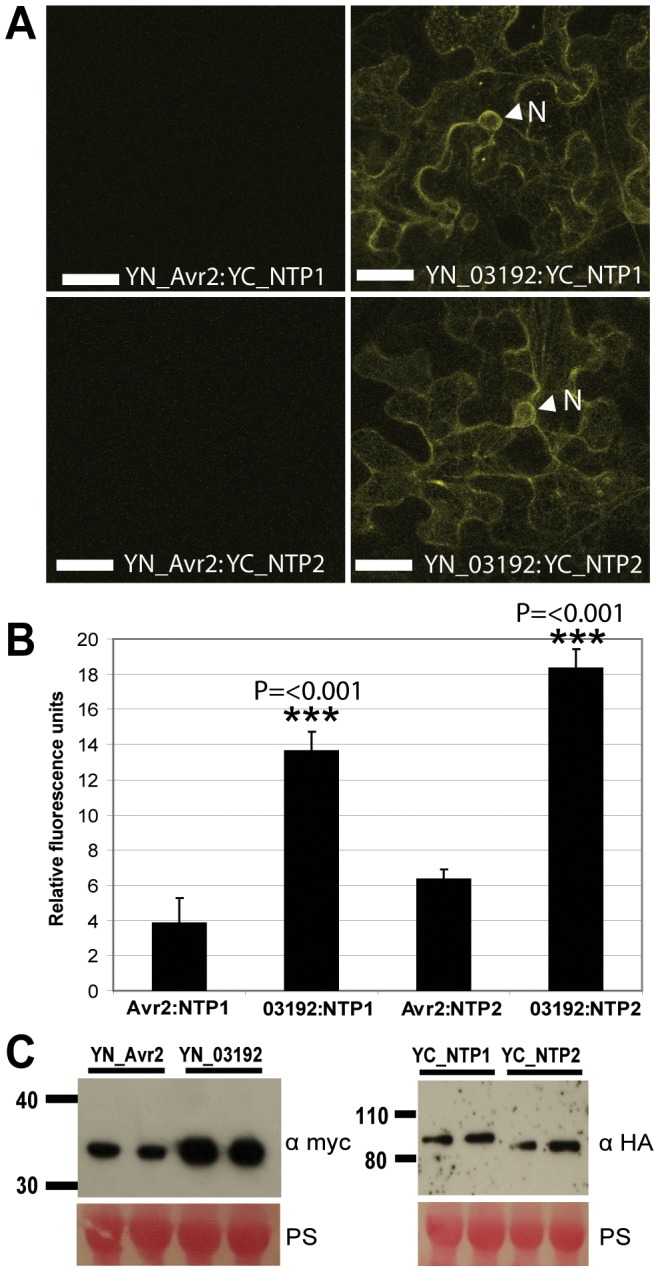
Interactions between Pi03192 and StNTP1 or StNTP2 occur at the ER membrane. (A) BiFC images showing that YFP fluorescence is reconstituted at the ER when YC-StNTP1 and YC-StNTP2 are co-expressed with YN-Pi03192, but not with YN-Avr2. The arrowheads indicated the ER membrane surrounding the nucleus. Scale bars indicate 35 µm. (B) Graph shows relative fluorescence quantified using a fluorimeter. Error bars show standard error and asterisks indicate a very highly significant difference (p<0.001, *t*-test) in fluorescence of YC-StNTP1 or YC-StNTP2 co-expressed with YN-Pi03192, compared to co-expression with YN-Avr2. (C) Immunoblots showing stability of full length YN-Pi03192 and YN-Avr2 (myc antibody) fusions and YC-StNTP1 and YC-StNTP2 (HA antibody) fusions.

To investigate further the potential interaction between Pi03192 and either StNTP1 or StNTP2 *in planta*, bimolecular fluorescence complementation (BiFC) was utilised. The non-interacting *P. infestans* RxLR effector PiAVR2 was used as a negative control, as it has been shown using BiFC to interact in the host cytoplasm with a different plant protein, StBSL1 [26]. These constructs were co-expressed in *N. benthamiana* plants and examined using confocal microscopy. YFP fluorescence was observed when YN-Pi03192 was co-expressing with either YC-StNTP1 or YC-StNTP2. However, no noticeable fluorescence was observed when co-expressing either YC-StNTP1 or YC-StNTP2 with YN-PiAVR2 (Figure 4A). The fluorescence of YN-Pi03192 co-expressed with either YC-StNTP1 or YC-StNTP2 was observed to be at the ER (Figure 4A, Figure S5), showing that they are in close proximity in this location, consistent with co-localisation of these proteins (Figure 3). Fluorescence was subsequently quantified in leaf disks co-expressing each construct, and showed significantly increased fluorescence (P≤0.001, One way ANOVA) when co-expressing YC-StNTP1 or YC-StNTP2 with YN-Pi03192, compared to YC-StNTP1 or YC-StNTP2 with YN-PiAVR2 (Figure 4B). Immunoblots of each construct used for SplitYFP show that all fusion proteins were stable *in planta* and of the predicted size (Figure 4C). Therefore, protein instability does not account for the reduced SplitYFP fluorescence observed when using PiAVR2, as opposed to Pi03192.

### Silencing of *NbNTP1* and *NbNTP2* in *Nicotiana benthamiana* enhances susceptibility to *P. infestans*

As Pi03192 was found to enhance *P. infestans* colonisation of the model host *N. benthamiana* (Figure 1), *N. benthamiana* was employed further to explore the roles of *NTP1* and *NTP2* in defence responses to this pathogen. Initially, PCR primers designed to amplify full-length *StNTP1* and *StNTP2* from potato (Supplementary Table S1) were used to amplify homologous genes from *N. benthamiana*. This resulted in two genes with 86% and 87% identity at the nucleotide level, respectively, to *StNTP1* and *StNTP2*. These sequences were annotated in the *N. benthamiana* genome [39] as NbS00058586g0005.1 and NbS00026810g0011.1. NbS00058586g0005.1 was found to be the reciprocal best BLAST match to *StNTP1* in the *N. benthamiana* genome, and was thus renamed *NbNTP1*; similarly, NbS00026810g0011.1 was the reciprocal best BLAST match to *StNTP2*, and renamed *NbNTP2*. The NAM domains of NbNTP1 and NbNTP2 protein sequences are placed in the same clades as StNTP1 and StNTP2, respectively, in our phylogenetic reconstruction (Figure S1–S3).

To assess the effects of NbNTP1 and NbNTP2 on basal resistance to *P. infestans*, two independent Virus Induced Gene Silencing (VIGS) constructs were designed to specifically silence each gene independently in *N. benthamiana*. Different portions of the *NbNTP* genes were cloned in antisense into the pTRV2 Agro binary vector [40] to create silencing constructs pTRV-NTP1 I and II, and pTRV-NTP2 I and II (Figure S6). *Agrobacterium* strains containing these constructs were infiltrated into 2-week-old *N. benthamiana* seedlings, and plants were left for 2-to-3 weeks to allow silencing to become systemic. Gene silencing levels were checked using quantitative real time (qRT)-PCR. Both pTRV-NTP1 VIGS constructs were observed to knock down *NbNTP1* expression by 80–90% and pTRV-NTP2 constructs knocked down *NbNTP2* expression by 70–90% compared to gene expression in the TRV-GFP control plants (Figure S6) Both pTRV-NTP1 VIGS constructs did not alter *NbNTP2* transcript levels, and pTRV-NTP2 constructs did not alter *NbNTP1* transcript levels. To investigate effects of silencing at the protein level, GFP-StNTP1 and GFP-StNTP2 fusions were transiently expressed in the *NTP1* and *NTP2* silenced plants and the levels of the GFP fusion proteins were examined by immunoblot analyses. Using a GFP antibody GFP-StNTP2 but not GFP-StNTP1 could be strongly detected in *NTP1*-silenced plants. In contrast, GFP-StNTP1, but not GFP-StNTP2 protein was strongly detected in *NTP2*-silenced plants (Figure S6). This demonstrates that the constructs designed to silence *NbNTP1* also silence *StNTP1*, but not *StNTP2*, whereas the constructs designed to silence *NbNTP2* also silence *StNTP2*, but not *StNTP1*. Moreover, reduction in transcript levels was seen to lead to specific reduction in levels of the corresponding protein.

**Figure 5:**
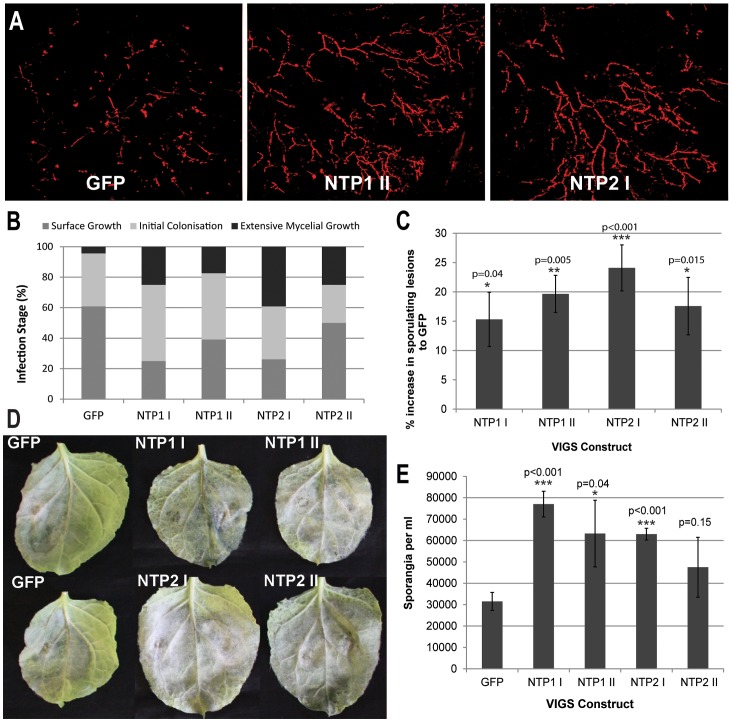
VIGS of *NbNTP1* and *NbNTP2* increases susceptibility to *P. infestans*. (A) Typical confocal images of *P. infestans* 88069-tdT growth (observed as Td-tomato fluorescence) at 3 dpi in unsilenced (GFP) or silenced (NTP1 II or NTP2 I as examples) for comparison with categories of infection in Figure S7). (B) Graph shows the percentage of *P. infestans* 88069-tdT infection sites on plants that are unsilenced (GFP) or expressing each VIGS construct (as indicated) which belong to each infection category (surface growth, initial colonisation, extensive mycelial growth) as visualised by confocal microscopy at 3 dpi. The results are combined data for 2 biological reps (n = 8 per construct per rep). (C) Graph shows the percentage increase in the number of inoculation points that are sporulating at 7 dpi, with the levels of sporulation in the GFP control set to zero. Error bars are standard error and the graph represents the combined data from 10 biological reps (n = 32 per construct per rep). Significant differences to the GFP control by *t*-test are indicated by asterisks and p values. (D) Photographs of leaves from the indicated VIGS plants infected with WT *P. infestans* at 10 dpi. (E). Sporangia per ml recovered at 10 dpi from each VIGS line. Error bars are standard error and significant difference to the GFP control by *t*-test are indicated by asterisks and p values.

Having shown that both sets of TRV-NTP1 and TRV-NTP2 VIGS constructs silence *NTP1* and *NTP2*, respectively, in *N. benthamiana*, we investigated whether *P. infestans* colonisation was enhanced by silencing them. Plants expressing these VIGS constructs, or the TRV-GFP control construct, were infected with a transgenic *P. infestans* strain expressing tandem dimer Tomato (tdT) [26]. TdT fluorescence was examined by confocal microscopy. We noted that colonisation was significantly delayed in TRV-GFP control plants, compared to plants lacking TRV (Figure S7A). At early stages of infection (3 dpi), extensive intercellular mycelial growth indicative of spreading lesions was observed on TRV-NTP1 and TRV-NTP2 plants at 20–40% of inoculation sites. In contrast, at 3 dpi on TRV-GFP control plants infections were largely restricted to leaf-surface growth of germinating sporangia and, in some cases, production of invasive hyphae indicative of initial pathogen colonisation (Figure 5A and B, Figure S7B). Thus, silencing either *NTP1* or *NTP2* promoted more rapid initial colonisation of *N. benthamiana* by *P. infestans*.

The increase in susceptibility following TRV-NTP-mediated VIGS was quantified by counting the percentage of *P. infestans* drop inoculation sites that showed sporulating lesions at 7 dpi compared to the TRV-GFP control, which was used as a base line. Figure 5C shows an increase of between 10–20% in the number of *P. infestans* sporulating lesions on the TRV-NTP VIGS plants compared to the TRV-GFP control (P<0.05; two tailed t-test). Figure 5D shows that, by 10 dpi, more *P. infestans* growth and sporulation was visible in *NTP1-* and *NTP2*-silenced plants than in the TRV-GFP-expressing control. The increase in susceptibility to *P. infestans* in TRV-NTP VIGS plants was measured at 10 dpi by quantifying sporangia harvested from leaves expressing each construct. Significantly more sporangia were recovered from TRV-NTP silenced plants than the TRV-GFP control plants (Figure 5E). The increase in susceptibility of *NTP1* and *NTP2* silenced plants to *P. infestans* infection suggests that these genes are involved in conferring basal resistance to this pathogen. Therefore, it seems logical for *P. infestans* to produce effectors, such as Pi03192, to interfere with the functions of these proteins.

### Silencing of *Pi03192* in *P. infestans* compromises pathogenicity which can be restored by transient *in plant* expression of Pi03192, and significantly enhanced on *NTP*-silenced plants

**Figure 6:**
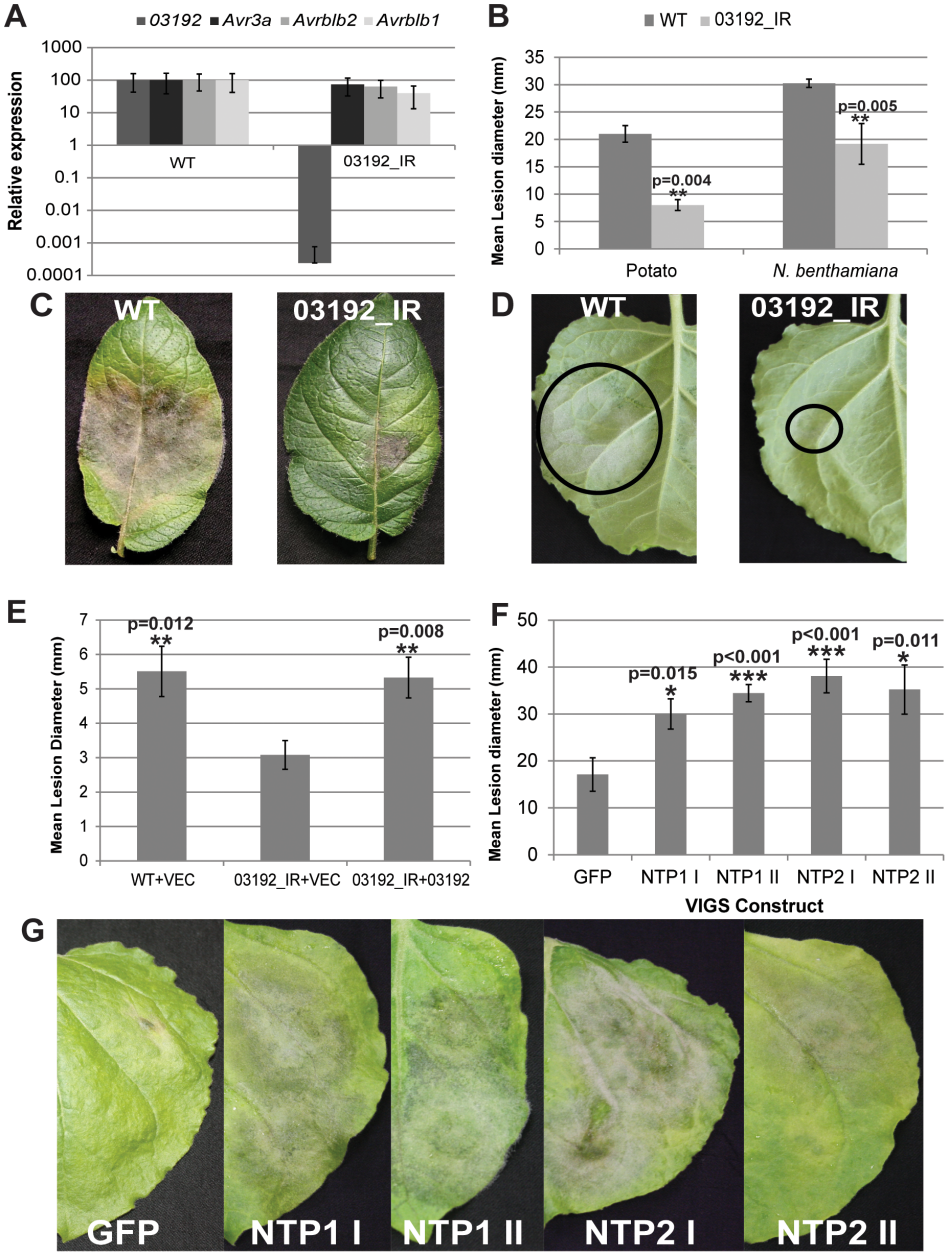
Silencing of *Pi03192* compromises pathogenicity which can be restored in TRV:NTP plants. (A) Graph shows expression levels of *Pi03192* are severely down regulated in 03192_IR compared to WT (strain 88069) while three other known *P. infestans* avirulences are unaffected. (B) Graph shows 03192_IR is significantly less able to infect both potato and *N. benthamiana* compared to WT (two tailed *t*-test p<0.005,n = 8). Images show symptoms of WT and 03192_IR infection on (C) potato and (D) *N. benthamiana*. (E) Graph shows that *in planta* expression of Pi03192 is able to restore virulence of 03192_IR to WT levels (strain 88069 inoculated with transient expression of empty vector control; vec) (no significant difference with one way anova p = 0.83, n = 55). In contrast, colonisation by 03192_IR with expression of the empty vector control (vec) is significantly reduced (one way anova p<0.05, n = 73). (F) Graph shows significant increases in lesion diameter for 03192_IR on each TRV:NTP silenced line compared to the TRV:GFP control (two tailed t-test p<0.05, n = 30). (G) Images show 03192_IR symptoms on TRV:NTP silenced plants and the TRV:GFP control. Error bars are standard error.

To examine the role of Pi03192 in *P. infestans* virulence a stably silenced transgenic line, hereafter referred to as 03192_IR, was made by transforming strain 88069 with the *Pi03192* gene cloned as an inverted repeat. QRT-PCR was employed to determine the levels of silencing in germinating cysts. Figure 6A shows that 03192_IR is specifically silenced for *Pi03192* expression and that the transcript accumulation of other RXLR genes with published roles in *P. infestans-*host interactions (*AVR3a*, *AvrBlb2*, and *AvrBlb1 [ipiO1]*) [24], [27], [30] is unaffected. Compared to wildtype *P infestans* isolate 88069, silenced line 03192_IR is less able to colonise both potato and *N. benthamiana* plants (Figure 6B–D) and forms both fewer and smaller lesions, indicating that Pi03192 function is important for full pathogenicity on these host plants. In *N. benthamiana*, colonisation by the 03192_IR line was restored to wild-type levels by transient agro-expression of Pi03192 inside host cells, compared to an empty vector control (Figure 6E).

When 03192_IR was inoculated onto either *NTP1* or *NTP2* silenced plants a significant increase in virulence was observed compared to growth on TRV::GFP controls. Figure 6F–G shows a significant increase in lesion size and increased symptom development, respectively, of 03192_IR on *NTP1* and *NTP2* silenced plants. This supports further the interactions of both NTP1 and NTP2 with Pi03192 and suggests Pi03192 exerts its virulence function by interfering with both NTP proteins, as silencing of either *NTP1* or *NTP2* using VIGS enhances pathogenicity of the compromised 03192_IR isolate.

### *NbNTP1 and NbNTP2* transcripts accumulate differentially in response to *P. infestans* infection versus treatment with culture filtrate from *in vitro* grown *P. infestans*

**Figure 7:**
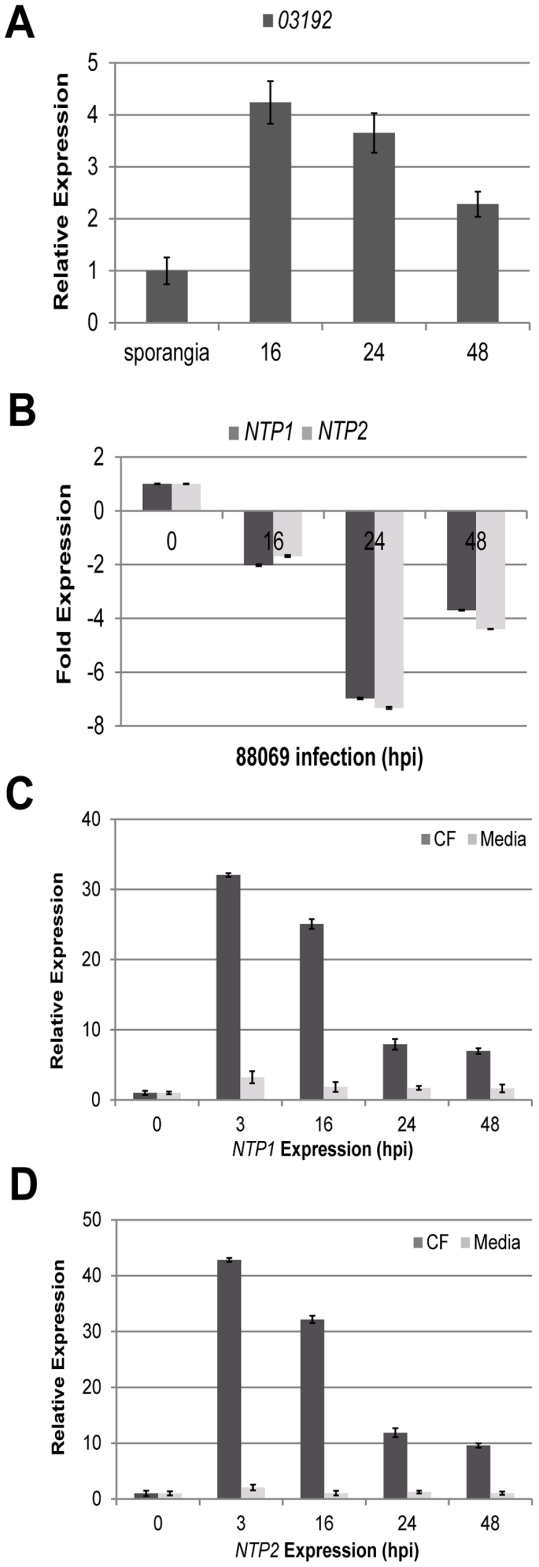
Differential transcript accumulation of *NbNTP* genes. Transcript abundance of (A) *Pi03192* (relative to expression in sporangia, which is given a value of 1) and (B) *NbNTP1* and *NbNTP2* (relative to uninoculated plant, which is given a value of 1) at 16, 24 and 48 hours post-inoculation of *N. benthamiana* plants infected with wildtype *P. infestans*. Error bars are standard error. Expression of (C) *NbNTP1* and (D) *NbNTP2* at 3, 16, 24 and 48 hours post-inoculation with either culture media or *P. infestans* culture filtrate (CF), relative to untreated plant (0). Error bars are standard error.

QRT-PCR was used to investigate expression of *NbNTP1* and *NbNTP2* across time-courses of *P. infestans* infection. *Pi03192* expression was measured during *P. infestans* infection of *N. benthamiana* and found to be up-regulated at 16 and 24 hours post-inoculation (hpi) compared to the levels of expression in sporangia samples (Figure 7A). The observed expression at 16 and 24 hpi time-points are consistent with previous observations of up-regulation of RXLR effector genes, generally, and *Pi03192* specifically, during potato and tomato colonisation [14], [19] and coincide with initial host cell penetration and formation of haustoria during the biotrophic phase [41].

QRT-PCR analysis of the same time-course samples showed that transcript accumulation of *NbNTP1* and *NbNTP2* co-ordinately decreased 2- to 6-fold in response to *P. infestans* infection (Figure 7B). The expression levels of *Pi03192*, *StNTP1* and *StNTP2* showed similar patterns during potato-*P. infestans* infection, demonstrating that the transcription of effector and *NTP* genes responds similarly in each pathosystem (Figure S8).

We next investigated expression of *NbNTP1* and *NbNTP2* after infiltration of *N. benthamiana* leaves with culture filtrate (CF) from *in vitro-*grown *P*. *infestans*, which we selected because it potentially contains a range of *Phytophthora* PAMPs. To confirm this, qRT-PCR analysis was conducted on a range of PTI marker genes in *N. benthamiana* treated with either flg22 or *P. infestans* CF. The marker genes *NbPTI5* and *NbACRE31* [42] and *NbWRKY7* and *NbWRKY8* [43] showed similar patterns of up-regulation in response to either flg22 or CF treatments (Figure S9), consistent with our hypothesis that *P. infestans* CF likely contains *Phytophthora* PAMPs. The lower levels of up-regulation of these PTI marker genes with CF is likely because the *P. infestans* molecules acting to trigger this response are highly diluted compared to the defined flg22 peptide treatment. Similar to *NTP1* and *NTP2*, transcript accumulation of all 4 PTI marker genes decreased significantly during infection (Figure S9).

In contrast to the observed decrease in *NTP* expression during infection, both *NbNTP1* and *NbNTP2* show coordinate 30- to 40-fold increases in transcript abundance measured at 3 hpi, with mRNA levels declining thereafter through the 48 hour time-course when the plant was treated with *P. infestans* CF (Figure 7C and D). Treating the plant with the media used to grow *P. infestans* for CF preparation did not induce *NbNTP* expression (Figure 7C and D). Interestingly, *NbNTP1* and *NbNTP2* transcripts failed to accumulated upon flg22 treatment (Figure S10). Taken together, these expression profiles distinguish flg22 and CF treatments, and suggest that *NbNTP1* and *NbNTP2* are up-regulated by a *P. infestans*-derived molecule in CF which activates a pathway that differs from the flg22/FLS2 PTI pathway. Both *NbNTP* genes show a similar pattern of expression in the different treatments, suggesting that they are co-ordinately regulated.

### Pi03192 prevents CF-triggered re-localisation of GFP-StNTP1 and GFP-StNTP2 from the ER into the nucleus

Membrane-bound NAC transcription factors (NAC-MTFs) are often associated with rapid transcriptional changes in response to biotic and abiotic challenges, due to stress-triggered cleavage of the TM domain, allowing the active TF to re-localise to the nucleus [35], [38]. As StNTP1 and StNTP2 have an ER membrane localisation and show elevated mRNA abundance in response to CF treatment, we hypothesised that application of CF would trigger their release from the ER membrane and lead to nuclear accumulation. To test the impact of Pi03192 effector on nuclear re-localisation of StNTP proteins, either a pFlub empty vector control (Vec) or pFlub-Pi03192 were co-expressed with either GFP-StNTP1 or GFP-StNTP2. The pFlub vector contains a dual cassette expressing an untagged gene and an RFP-peroxisome tagged construct, which allows visualisation of cells (i.e. containing peroxisomes tagged with RFP) that also express an untagged gene of interest, in this case *Pi03192*.

**Figure 8:**
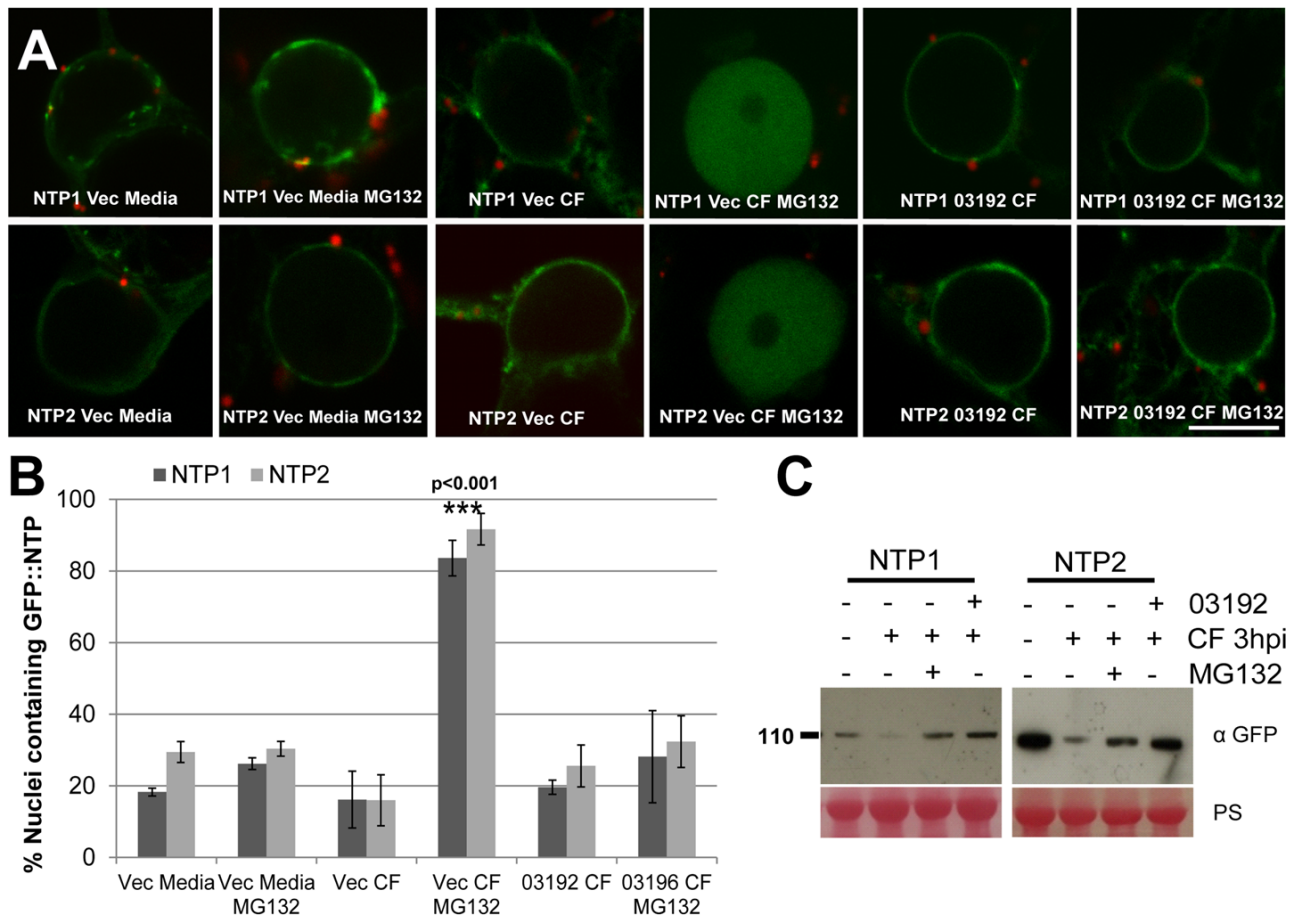
Pi03192 prevents CF-mediated accumulation of StNTP1 and StNTP2 in the nucleus. (A) Confocal images of localisation of either GFP-StNTP1 or GFP-StNTP2 co-expressing either pFlub (vec) or pFlub-03192 and treated with Media or culture filtrate, plus and minus MG132. Scale bar is 10 µm. (B) Graph shows the percentage of nuclei containing either GFP-StNTP1 or GFP-StNTP2 fluorescence with each treatment. Error bars are standard error and one-way ANOVA analysis shows that treatment with CF and MG132, when co-expressed with empty vector (vec), is statistically different from the other treatments, which are not different from each other. Significance is indicated by p-value and asterisks. (C) Immunoblots show the stability of GFP-StNTP1 and GFP-StNTP2 with different treatments as indicated, probed with a specific GFP antibody. PS is Ponceau stain.

*N. benthamiana* plants co-expressing either GFP-StNTP1 or GFP-StNTP2 with pFlub (empty vector control) were treated with either media or CF and examined using a confocal microscope. No change in localisation of fluorescence was observed, and both treatments showed GFP fluorescence associated only with the ER membrane surrounding the nucleus, albeit fluorescence was reduced following CF treatment (Figure S11). Accumulation of nuclear GFP-StNTP fluorescence was not evident following CF treatment (Figure 8A). However, earlier experiments with the GFP-StNTPΔTM fusions demonstrated that nuclear NTP1 and NTP2 are turned over by the 26S proteasome (Figure S4). Therefore, samples were simultaneously treated with CF and proteasome inhibitor MG132 to block degradation. Confocal images following treatment with both CF and MG132 showed accumulation of both GFP-StNTP1 and GFP-StNTP2 inside the nucleus. In contrast, treatment with the medium used to grow *P. infestans* for CF preparation, alongside MG132, did not result in nuclear accumulation of either StNTP protein (Figure 8A). Moreover, whereas CF treatment with MG132 resulted in significant accumulation of GFP-NTP1 and GFP-NTP2 in nuclei, no such accumulation was observed following flg22 treatment with MG132 (Figure S12). This demonstrates that *P. infestans* CF treatment provides a specific trigger, which differs from that which activates the flg22/FLS2 PTI pathway, to release StNTP proteins from the ER membrane and allow their translocation inside the nucleus, after which they are turned over through the action of the 26S proteasome.

To examine the effect of the RXLR effector Pi03192 on this process, leaves of *N. benthamiana* were infiltrated with *Agrobacterium* strains co-expressing GFP-StNTP1 or GFP-StNTP2 with pFlub-Pi03192. Confocal images of these samples, treated with either CF alone or CF plus proteasome inhibitor MG132, show that GFP-StNTP1 and GFP-StNTP2 fluorescence no longer accumulates inside the nucleus. Instead, even with MG132 treatment, GFP-StNTP1 and GFP-StNTP2 fluorescence was only detected in association with the ER, consistent with the interpretation that Pi03192 prevents release of GFP-StNTPs from the ER membrane (Figure 8A).

To confirm this result, a quantitative analysis was carried out on a series of confocal images of samples subjected to each of the treatments where the percentage of nuclei containing GFP-StNTP fluorescence was recorded. Figure 8B shows that the percentage of nuclei containing GFP-StNTP1 or GFP-StNTP2 fluorescence, co-expressed with empty pFlub vector and treated with CF plus MG132, is significantly increased compared to all other treatments (P≤0.001), which do not differ significantly from each other.

Immunoblots were performed to assess turnover of StNTP1 and StNTP2 when treated with CF, in the presence or absence of MG132 or Pi03192. Figure 8C shows that, when co-expressed with the pFlub vector control, the detection of both GFP-StNTP1 and GFP-StNTP2 decreases upon CF treatment, consistent with the observed reduction in GFP fluorescence (Figure S11). This is likely due to release of the StNTP protein from the ER and turnover by the proteasome, whereas turnover is prevented in the presence of MG132. Critically, when treated with CF, turnover of GFP-StNTP1 and GFP-StNTP2 is also prevented by co-expression with Pi03192 even in the absence of MG132 (Figure 8C).

Our results suggest it is likely that Pi03192 prevents release of StNTP1 and StNTP2 from the ER, rather than the effector preventing entry of these TFs into the nucleus. To further investigate this, we co-expressed pFlub vector (control) or pFlub-Pi03192 with GFP-StNTP1ΔTM or GFP-StNTP2ΔTM, and treated with MG132. Pi03192 did not prevent nuclear accumulation of either GFP-StNTPΔTM fusion protein, indicating that the effector does not prevent their entry into the nucleus (Figure S13). We conclude that release of StNTP1 and StNTP2 from the ER, leading to them entering the nucleus, is prevented by the action of Pi03192.

## Discussion

Little is known about the identities of host proteins that are targeted by effectors from filamentous plant pathogens such as oomycetes and fungi. Less is known about the roles those targets play in plant immunity, or other roles detrimental to disease progression, and less still about the modes of action of effector proteins upon such targets. Here we show that the *P. infestans* RXLR effector Pi03192 enhances infection when expressed transiently inside *N. benthamiana* cells. Pi03192 interacts with the C-terminal portions of two ER-associated potato NAC transcription factors, called StNTP1 and StNTP2. Interaction *in planta* occurs at the ER. VIGS of the equivalent *NbNTP1* and *NbNTP2* genes in *N. benthamiana* leads to accelerated biotrophic colonisation, and increased sporulation in later stages of infection by *P. infestans*, compared to unsilenced plants. A stable transgenic *P. infestans* line (03192_IR) silenced for *Pi03192* expression shows decreased virulence on both potato and *N. benthamiana*, indicating that the effector has a non-redundant function and is essential for full virulence. Virulence of the 03192_IR line was restored to WT levels by transient *in planta* expression of Pi03192, and was significantly enhanced on *N. benthamiana* plants that were VIGSed to knock down expression of either *NTP1* or *NTP2*, indicating that knock-down of an essential virulence component can be complemented by knock-down of its target(s). Critically, we provide evidence that the mode-of-action of Pi03192 is to prevent PAMP-triggered re-localisation of StNTP1 and StNTP2 from the ER into the nucleus. Each of these observations is discussed below.

Thirteen membrane-associated NAC TFs with putative C-terminal transmembrane (TM) domains [35], [44], termed NTM1-like (NTL) proteins, have been reported in Arabidopsis. Members of this family contribute rapid responses to biotic and abiotic stresses. NTL4 promotes production of reactive oxygen species in response to drought [45]. NTL8 is involved in GA-mediated signalling following salt stress [46]. NTL6 promotes SA-independent activation of defences, including up-regulation of *PR* genes, upon cold treatment [47]. NTL11 (also called RPX) has been shown to positively regulate the 26S proteasome [48] and NTL9 regulates leaf senescence in response to osmotic stress [49]. We find from searches of the RefSeq sequence database that there are more than 13 *NTL* genes (proteins predicted to contain at least one NAM domain, and a transmembrane domain at the C-terminal region) in *A. thaliana* (Figure S1 and S2). NTL9 was found to be a common interacting protein of RXLR effectors from *Hyaloperonospora arabidopsidis* (*Hpa*) and T3SS effector HopD1 from *Pseudomonas syringae* pv. *tomato*. Knockout of *NTL9* in *A. thaliana* resulted in increased susceptibility to *Hpa* [23].

Comparison of NAM domains indicates that StNTP1 and three other potato NAC proteins with predicted TM domains are placed in the same clade as Arabidopsis NTL6, while StNTP2 and a further three potato NAC proteins with predicted TM domains are found in a clade with Arabidopsis NTL1, NTL3 and NTL7 (Figures S1, S2). Both of these placings have exceedingly strong bootstrap support. It is not, however, possible to associate either StNTP1 or StNTP2 with a specific counterpart protein in Arabidopsis on the basis of this tree. NAM domains are expected to bind to specific regions of DNA, and so express the regulatory component of NAC TF function. Reconstruction of phylogeny on the basis of this protein domain may therefore reflect conservation of regulatory targeting, as the evolution of NAM domains will be constrained by the need to bind specific DNA sequence sites. Approximately half of the 337 sequences used to construct the tree were predicted to have TM domains at their C-terminus. Several clades containing sequences from a wide range of plant species are homogeneous for the presence of such a predicted domain. This suggests a general conservation across plant species of both a specific nuclear DNA target, and membrane association that is possibly a mechanistic requirement for rapid regulatory response by release from subcellular membranes. We note that the reconstructed phylogeny decomposes into a set of 40 distinct clades with 100% bootstrap support at their distal node, but that each such clade may contain multiple proteins from the same plant, either with or without a predicted TM domain. Most such clades contain either monocot or dicot plant proteins, but not both. Several clades contain a notable overrepresentation of proteins from Arabidopsis, with paired sequences from *A. thaliana* and *A. lyrata*, suggesting an expansion of this family in those species. Further work beyond the scope of this current study is required to determine experimentally whether proteins with similar NAM domains share common or overlapping promoter binding sites, or regulate expression of similar gene targets.

Both *NbNTP1* and *NbNTP2* genes were co-ordinately up-regulated by treatment with CF from *in vitro* grown *P. infestans*, which we demonstrate activates early PTI-responsive marker genes in *N. benthamiana*. We therefore infer that CF potentially contains a mixture of *Phytophthora* PAMPs. Nevertheless, *NbNTP1* and *NbNTP2* transcripts did not accumulate following treatment with the defined PAMP, flg22. Thus, up-regulation of *NbNTP1* and *NbNTP2* follows perception of a *P. infestans*-derived molecule in CF which activates a pathway independent of the FLS2/flg22 PTI pathway.

During *P. infestans* host colonisation, transcript abundance of *NbNTP1* and *NbNTP2* (Figure 7), and of the PTI marker genes *NbPTI5* and *NbACRE31* [42] and *NbWRKY7* and *NbWRKY8* [43] (Figure S9), was sharply reduced, indicating that the active presence of the pathogen counteracts their induction. This indicates that the pathogen actively suppresses PTI. Moreover, the down-regulation of *NbNTP1* and *NbNTP2* indicates the potential contribution of these NAC TFs to preventing late blight disease.

VIGS of either *NbNTP1* or *NbNTP2* resulted in enhanced susceptibility to *P. infestans*, confirming that their protein products reduce disease progression (Figure 5). The fact that VIGS of either *NbNTP1* or *NbNTP2* led to enhanced *P. infestans* colonisation implies that their roles in immunity are either functionally non-redundant, or that they act together, perhaps as heterodimers, and are thus inter-dependent. Hetero- and homo-dimerisation of NAC TFs has been demonstrated [50], and we observe that the sites for dimerization are conserved in the NAM domains of both StNTP1 and StNTP2 (Figure S3) although the contribution of the C-terminal domains is not clear. It is therefore at least plausible that these proteins act as a heterodimer, and this may explain why VIGS of either *NbNTP1* or *NbNTP2* alone enhances *P. infestans* colonisation.

A transgenic *P. infestans* line was generated in which *Pi03192* was silenced, but not other effectors which have demonstrated roles in virulence, such as *AVR3a*, *AvrBlb1*, and *AvrBlb2* [24], [27], [30]. Remarkably, the virulence of this silenced line, which was compromised on both potato and *N. benthamiana*, was significantly enhanced on the latter not only when Pi03192 was transiently expressed in plant cells, but also when either *NbNTP1* or *NbNTP2* were silenced by VIGS. The restoration of virulence following VIGS of either gene again suggests that the roles of NTP1 and NTP2 may be inter-dependent, or overlap, perhaps explaining why both are targeted by the Pi03192 effector.

A NAC TF called TIP has been reported to interact with the coat protein of *Turnip Crinkle Virus* (TCV), and this interaction was required to elicit the hypersensitive response triggered by HRT resistance protein [51]. GFP-TIP was found to localise to the nucleus in the absence of TCV. Notably, the presence of TCV coat protein resulted in exclusion of TIP from the nucleus, compromising its ability to regulate defence responses to TCV [52]. The authors postulated that exclusion of TIP from the nucleus by TCV coat protein was detected by HRP [52]. This indicates the potential for pathogens to interfere with the appropriate localisation of NAC TFs, and for the plant to ‘guard’ against such activity.

To investigate the mode-of-action of Pi03192, we studied re-localisation of StNTP1 and StNTP2 into the nucleus following treatment with *P. infestans* CF. As both TFs are turned over in the nucleus by the 26S proteasome, these experiments were conducted in the presence of the proteasome inhibitor MG132. We showed that treatment with CF and MG132 resulted in a clear, measurable accumulation of GFP-StNTP1 and GFP-StNTP2 fluorescence in the nucleus. No such accumulation was observed following co-treatment with MG132 and the medium used for *P. infestans* growth to prepare CF, or by treatment with flg22, demonstrating that CF contains a specific trigger from the pathogen that causes release of NTP1 and NTP2 from the ER in order to enter the nucleus. Critically, in the presence of Pi03192, no increase in nuclear accumulation of GFP-StNTP1 or GFP-StNTP2 was observed following CF treatment, even when these plants were also treated with MG132 (Figure 8). GFP fluorescence was instead confined to the ER, indicating that the effector has either prevented release of the NTP TFs, or has prevented their entry into the nucleus. The observation that Pi03192 did not prevent accumulation of GFP-StNTP1ΔTM or GFP-StNTP2ΔTM following MG132 treatment, suggests that the effector does not inhibit entry into the nucleus. Moreover, the original Y2H screen revealed that Pi03192 interaction was maintained with just the C-termini of StNTP1 and StNTP2, containing the TM domains. We conclude that Pi03192 prevents release of the NTP TFs from the ER.

Immunoblots of the critical experiments showed that, in the presence of CF alone, the detection of both GFP-StNTP1 and GFP-StNTP2 was reduced, consistent with the 26S proteasome acting to degrade them when they are released from the ER to enter the nucleus. In agreement with this, treatment with both CF and MG132 restored stability of GFP-StNTP1 and GFP-StNTP2. Crucially, in the presence of Pi03192 both GFP-StNTP1 and GFP-StNTP2 were strongly detected in the immunoblot following treatment with CF alone, showing that stability is restored by the effector in the absence of MG132. Previous studies of *Arabidopsis* NTLs have shown that they are proteolytically cleaved from the ER so that they can enter the nucleus [35], [37], [44]. We were, however, unable to detect smaller products from GFP-StNTP1 and GFP-StNTP2 when treated with both CF and MG132. It is possible that cleavage is sufficiently close to the TM domain, which represents only the last 18–29 amino acids of the C-termini of each, respectively, so as not to alter detectably the size of these proteins on a gel. Nevertheless, the failure to detect fluorescence from either of the GFP-NTP TFs in the nucleus when co-expressed with Pi03192 and treated with both CF and MG132 demonstrates that the effector has acted to prevent their entry into the nucleus where they can be turned over by the proteasome.

The nucleus is a centre of activity for both PTI and ETI, and many critical regulators of either are trafficked there from various subcellular locations following pathogen perception [32]–[33]. However, few plant pathogen effectors have been shown to influence such re-localisation events and, indeed, few have been shown to target transcriptional regulators of plant immunity. Within the nucleus itself, host transcriptional reprogramming has been shown to be affected by transcriptional activator-like (TAL) effectors from *Xanthomonas* spp, whereas the type III effector XopD interacts directly with the host TF AtMYB30 to prevent its activity [reviewed in 32–33]. Here, we show that the *P. infestans* RXLR effector Pi03192 targets two NAC proteins, NTP1 and NTP2, and prevents them from being released from the ER to enter the nucleus, where they contribute to prevent disease progression by this oomycete pathogen.

## Materials and Methods

### Yeast-2-hybrid assays

Y2H screening was performed using the ProQuest system (Invitrogen). Briefly, DNA binding domain “bait” fusions were generated by recombination between pDonr201-03192 and pDEST32, generating pDest32-03192. This construct was transformed into yeast strain MaV203, and nutritional selection used to recover transformants. A single transformant was grown up and used to prepare competent yeast cells, which then were transformed with a potato Y2H "prey" library, commercially prepared from *P. infestans* infected leaf material at 15 and 72 hpi. Candidate interacting preys were confirmed by retransformation with the 03192 bait construct or with a pDest32-AVR2 control to rule out the possibility that the observed reporter gene activation had resulted from interactions between the prey and the DNA binding domain of the bait construct or DNA binding activity of the prey itself.

### Plant material and microbial strains

*Nicotiana benthamiana* seedlings were grown in individual pots in a glasshouse at 22°C (16 hours light, 8 hours dark), with 130–150 µE m^−2^ s^−1^ light intensity and 40% humidity. *Agrobacterium tumefaciens* strain AGL1 virG was used for all *Agrobacterium* transient expression experiments and was cultured for 24–48 h in Luria Broth at 28°C supplemented with the appropriate antibiotics, spun at 4000 rpm and resuspended in 10 mM MgCl_2_∶10 mM MES buffer with 200 µM Acetosyringone to OD_600_ = 0.3 for Western Blot or 0.01–0.1 for confocal imaging.

### Cloning of protein fusions and VIGS constructs

Full length *StNTP* genes were cloned from potato cDNA with gene specific primers modified to contain the Gateway (Invitrogen) attB recombination sites. PCR products were purified and recombined into pDONR201 (Invitrogen) to generate entry clones via BP reactions using Gateway technology (Invitrogen). Pi03192 and PiAvr2 were cloned into pDONR201 in the same way. Primer sequences are shown in Supplementary Table S1. Protein fusions were made by recombining the entry clones with the following plant expression vectors using LR clonase (Invitrogen). N terminal GFP fusions were made by recombining the entry clones with pB7WGF2 [53]. Split YFP constructs were made by recombining the entry clones for Pi03192 and PiAvr2 with pCL112 to generate YN protein fusions and StNTP1 and 2 were recombined with pCL113 to generate YC protein fusions [24]. Pi03192 was recombined with pFlub4, a homemade variant of pMDC32 [54] where the Hyg^R^ cassette was replaced with a Peroxisome mRFP tagged expression construct. Virus induced gene silencing (VIGS) constructs were made by cloning 250 bp PCR fragments of *NTP1* and *2* from *N. benthamiana* cDNA and cloning into pBinary Tobacco Rattle Virus (TRV) vectors [40] between HpaI and EcoRI sites in the antisense orientation. A TRV construct expressing GFP described previously was used as a control [55]. Primer sequences are shown in Supplementary Table S1. The two largest leaves of four leaf stage *N. benthamiana* plants were pressure infiltrated with LBA4404 *A. tumefaciens* strains containing a mixture of RNA1 and each NAC VIGS construct or the GFP control at OD_600_ = 0.5 each. Plants were used for assays or to check gene silencing levels by qRT-PCR 3 weeks later.

### Confocal microscopy

*A. tumefaciens* containing each construct was pressure infiltrated into leaves of 4-week-old *N. benthamiana*. Cells expressing fluorescent protein fusions were observed using a Leica TCS-SP2 AOBS confocal microscope between one or two days post-infiltration. Images were obtained using an HCX APO 40×/0.9w water dipping lens. mRFP was imaged using an excitation wavelength of 568 nm from a ‘lime’ diode laser with emissions collected between 600 and 630 nm. GFP was imaged using 488 nm excitation from an argon laser, with emissions collected between 500 and 530 nm. Split-YFP was imaged using 514 nm excitation from an argon laser with emissions collected between 530–575 nm.Treatments were infiltrated 24 h after agro-infiltration of the constructs and leaves were observed under the confocal microscope 4 h later. Treatments are Media (Pea Broth), CF (*P. infestans* culture filtrate), MG132 (100 µM MG132 diluted in culture filtrate). For Pi03192 treatments GFP-NAC constructs were co-infiltrated with either pFlub-empty vector or pFlub-Pi03192 and subsequently treated with CF or MG132 as above. *P. infestans* strain 88069 expressing tandem dimer Tomato (tdT) fluorescent protein was imaged *in planta* using 10× dry lens and excited with 561 nm and emissions were collected between 570–600 nm using a lime diode laser.

### Fluorimeter assay

Quantification of fluorescence was performed using a SpectraMax M5 fluorimeter (Molecular Devices). Leaf disks were cut from *N. benthamiana* leaves at 2 dpi Agro-infiltration and floated abaxial side up on H_2_O in 24-well plates readings were taken using a well scanning program taking reads from above. Softmax Pro software (Molecular Devices) was used to collect data. YFP fluorescence was excited at 514 nm and measured at 580 nm. GFP fluorescence was excited at 480 nm and measured at 520 nm.

### SDS PAGE and Western analysis

Leaf disks were harvested at 2 dpi after *Agrobacterium* infiltration with constructs expressing either GFP-NAC1 or 2, GFP-Pi03192, YN_PiAvr2, YN_Pi03192, YC_NAC1, YC_NAC2 and were either untreated, or treated with one of the following: Buffer 3hpi (10 mM MgCl_2_∶10 mM MES), CF 3hpi (*P. infestans* culture filtrate), MG132 3hpi (100 µM MG132 diluted in culture filtrate). For Pi03192 treatments GFP-NAC constructs were co-infiltrated with either pFlub-empty vector or pFlub-Pi03192 and subsequently treated with CF 3hpi or MG132 3hpi as above. 1 cm^2^ leaf disks were ground in LN2 and resuspended in 100 ul 2X SDS PAGE sample loading buffer and loaded onto a 12% Bis-Tris NuPAGE Novex gel run with 1X MOPS SDS running buffer for 1.5 h at 120 V (Invitrogen). Gels were blotted onto a nitrocellulose membrane for 1.5 h at 30 V and stained with ponceau solution to show loading and transfer. Membranes were blocked in 5% milk in 1X PBS before addition of the primary antibodies at 1∶1000 dilutions: either a Monoclonal GFP antibody raised in mouse (Sigma-Aldrich), a Monoclonal Anti HA antibody raised in rabbit (Sigma-Aldrich) or a Monoclonal Anti MYC antibody raised in rabbit (Sigma-Aldrich). The membrane was washed with 1X PBST (0.2% tween 20) before addition of the secondary antibody at 1∶5000 dilution either Anti-Mouse IG HRP antibody (Sigma-Aldrich) or Anti-Rabbit IG HRP antibody (Sigma-Aldrich). SuperSignal West Femto (Thermo Scientific) ECL detection was used according to the manufacturer's instructions.

### Gene expression analysis

RNA was extracted using Tri Reagent [56]. RNA was treated to remove DNA contamination using a DNase Turbo Free kit (Ambion) according to the manufacturer's instructions. First strand cDNA was synthesised from 2 µg of RNA using Superscript II RNase HReverse Transcriptase (Invitrogen) according to manufacturer's instructions. Realtime qRT-PCR reactions were performed using Power SYBR Green (Applied Biosystems) and run on a Chromo4 thermal cycler (MJ Research, UK) using Opticon Monitor 3 software. Primer pairs were designed outside the region of cDNA targeted for silencing following the manufacturer's guidelines. Primer sequences in Supplementary Table S1. Detection of real-time RT-PCR products, calculations and statistical analysis were performed as previously described [57].

### *P. infestans* infection assay

*Phytophthora infestans* strains 88069 and an 88069-tdT transformant [26] were used for plant infection and were cultured on Rye Agar at 19°C for 2 weeks. Plates were flooded with 5 ml H_2_O and scraped with a glass rod to release sporangia. The resulting solution was collected in a falcon tube and sporangia numbers were counted using a haemocytometer and adjusted to 15000 sporangia/ml, 10 µl droplets were inoculated onto the abaxial side of detached *N. benthamiana* leaves stored on moist tissue in sealed boxes. For VIGSed plants the number of inoculated lesions which were sporulating at 7 dpi were counted and expressed as a percentage increase sporulating lesions compared to the GFP control plants. Sporangia counts were performed on 10 dpi leaves from VIGSed plants which had been immersed in 5 ml H_2_O and vortexed to release sporangia. A Haemocytometer was used to count the number of sporangia recovered from each leaf and was expressed as sporangia/ml. VIGSed plants inoculated with an 88069-tdT transformant were imaged using a confocal microscope at 3 dpi to examine P. infestans growth microscopically. *Agrobacterium tumefaciens* Transient Assays (ATTA) in combination with *P. infestans* infection were carried out as described [24], [26]. Briefly, *Agrobacterium* cultures were resuspended in agroinfiltration medium at a final concentration of OD600 = 0.1 and used for transient expression in planta by agroinfiltration. After 1 day, each infiltration site was inoculated with 10 µl zoospores from *P. infestans* isolate 88069 at 15000 sporangia/ml.

### Stable Silencing of *Pi03192* in *P. infestans*

An inverted repeat construct of *Pi03192* was cloned using primers indicated in Supplementary Table S1 in to the pSTORA vector and transformed into wildtype isolate 88069 as described previously [24]. The 03192_IR transformant was maintained on Rye agar plates supplemented with geneticin 20 µg/ml and gene expression levels from germinating cysts were determined by qRT-PCR as described previously [24], using primer sequences in Supplementary Table S1. *P. infestans* 03192_IR infection assays and ATTA experiments were performed as above.

### Phylogenetic reconstruction

The HMMer3 [58] hmmsearch package was used to query: the NCBI RefSeq/nr sequence database; the PGSC *Solanum phureja* genome annotation v3.4; the *Nicotiana benthamiana* NBGI.042210 release from the Dana Farber Cancer Institute; and *Solanum lycopersicum* annotations from the ITAG2.3 annotation and www.plantgdb.org (all downloaded 22/3/2012) for NAM domain-containing proteins, using the HMM profile for the NAM domain family (PF02365; http://pfam.sanger.ac.uk/family/PF02365) with the GA cutoff, producing a set of 2552 unique NAM domain protein sequences. These sequences were aligned using HMMer3's hmmalign package, and sequences with less than 40% sequence identity to any other sequence in the alignment discarded. The resulting set of 1700 sequences were clustered using MCL using an inflation value of 6.0, and a single cluster of 406 sequences containing the NAM domains of both StNTP sequences was retained. The aligned sequences were back-translated by threading against the draft genomes from the genome sequencing projects from which they derived, or against their corresponding entry in the NCBI nr database as appropriate, using the Python script (https://github.com/widdowquinn/scripts/blob/master/bioinformatics/get_NCBI_cds_from_protein.py), to give 374 aligned nucleotide sequences.

The nucleotide alignment was processed with trimal [59] to remove all columns containing more than 10% gaps, producing an alignment with 337 sequences and 374 characters. jModelTest [60] was used to determine an appropriate substitution model for phylogenetic reconstruction which, by consensus of AIC and BIC, was GTR+I. A maximum likelihood tree was produced for this alignment using raxML [61] with 100 bootstrap trees, using the GTRCATI model. The resulting tree was rendered with FigTree (http://tree.bio.ed.ac.uk/software/figtree/).

Transmembrane domains were predicted using the most complete available amino acid sequence available for each NAM domain-containing protein in the 2552 sequence set, using TMHMM v2.0 [62]. 161 NAM domain-containing proteins were predicted to contain a TM domain, of which 149 (92%) were also in the cluster of 406 sequences containing the StNTP proteins identified by MCL.

### Accession numbers

Pi03192 is PITG_03192 (GenBank accession: XM_002906231.1 GI: 301117097); StNTP1 (GenBank accession: KF437522); StNTP2 (KF437523); NbNTP1 (KF437524) and NbNTP2 (KF437525).

## Supporting Information

Figure S1 **Phylogenetic tree of plant NAC transcription factors.** Maximum Likelihood phylogenetic reconstruction for 337 NAM domains, extracted from plant NAC transcription factors. Red branches indicate NAC TFs that also possess a predicted C-terminal transmembrane domain. Clades are coloured by group for their originating plants: Arabidopsis (green; several shades are used for visual separation of clades), *Solanaceae* (yellow; orange used to indicate clades containing StNTP1 and StNTP2) and cereals (blue). Sequences are divided into 40 clades that each have 100% bootstrap support at the distal node. Of the previously classified AtNTL proteins, StNTP1 is most closely associated with AtNTL6, and StNTP2 equally with AtNTL1, AtNTL7 and AtNTL3. However, there is no clear one-to-one association of any StNTP with any AtNTL. Notable features include: the presence of many homogeneous TM domain-containing clades; the absence of grass sequences in the clades containing NTL proteins; and the apparent expansion of NAM domain-containing proteins in Arabidopsis.
(PDF)

Figure S2 **Phylogenetic tree of plant NAC transcription factors.** Maximum Likelihood phylogenetic reconstruction for 337 NAM domains, extracted from plant NAC transcription factors. This figure is identical to Figure S1, except for layout and the individual labelling of proteins. Proteins labelled in blue are Arabidopsis AtNTL proteins, and those labelled in red are predicted to contain a C-terminal transmembrane domain.
(PDF)

Figure S3 **Alignment of NAC DBDs for StNTPs and AtNTLs.** The NAC DNA binding (NAM) domains for the 13 published Arabidopsis NTLs were aligned with the NAM domains of both potato and *N. benthamiana* NTP1 and NTP2 proteins. Conserved residues are shaded black while those shaded grey share similar properties. Residues essential for DNA binding are marked with a # and those required for NAC dimerization are marked with *.
(PDF)

Figure S4 **Localisation and stability of delta TM NAC constructs.** A. Confocal images of GFP-StNTP1ΔTM and GFP-StNTP2ΔTM plus or minus MG132 treatment. The first two panels of each row are images with a x20 lens with scale bars representing 100 µm. The last panel of each row are images using a x64 lens zoomed in on a single nucleus with the scale bars representing 10 µm. B. Immunoblots of GFP-StNTP1ΔTM and GFP-StNTP2ΔTM plus or minus MG132 treatment probed with a specific GFP antibody, numbered ladder on the left represent size in kDa and PS is ponceau staining.
(PDF)

Figure S5 **Split YFP of ER localised interaction of Pi03192 and StNTPs.** Confocal images of YC-StNTP1 or YC-StNTP2 co-expressed with YN-Pi03192 showing clear ER localisation with inset slices showing the ER around the nucleus. Scale bars are 10 µm.
(PDF)

Figure S6 **NTP VIGS constructs and gene transcript and protein levels.** A. Schematic representations of the *NbNTP1* and *NbNTP2* genes showing the location of the region used to make each VIGS construct and the location of the qRT-PCR primers (arrows). B. Graph shows relative expression of the *NbNTP1* and *NbNTP2* genes in each VIGS line with the unsilenced (GFP) control set to 1. Error bars are standard error. C. Immunoblot showing the accumulation of GFP-StNTP1 and GFP-StNTP2 in unsilenced plants (GFP) and plants expressing each of the VIGS constructs as indicated, probed with a specific GFP antibody. PS is Ponceau stain. Sizes are indicated in kD.
(PDF)

Figure S7 **Early infection categories for** ***P. infestans*** **88069-tdT.** A: Graph shows the presence of TRV significantly reduces the rate of *P. infestans* colonisation compared to non TRV plants (two tailed *t*-test p<0.001, n = 6), error bars are standard error. B: Representative confocal images showing what each infection category looks like, as measured in Figure 5B. Surface growth shows sporangia, germinating sporangia and infection hyphae on the leaf surface. Initial colonisation shows the above but with the addition of some hyphal penetration of the tissue immediately below the site of inoculation. Extensive mycelial growth shows mycelial growth of hyphae through the leaf mesophyll layers extending beyond the site of inoculation.
(PDF)

Figure S8 **QRT-PCR of** ***P. infestans*** **/potato infection.** Expression of A. *Pi03192* and B. *StNTP1* and *StNTP2* at 24 and 48 hours post-inoculation of potato cv Bintje plants infected with wildtype *P. infestans*. Error bars are standard error.
(PDF)

Figure S9 **QRT-PCR of PTI marker genes following flg22 and culture filtrate treatments and during infection.** Relative expression, compared to untreated plants (0), of known PTI marker genes *NbPti5*, *NbAcre31*, *NbWRKY7* and *NbWRKY8* at 3 and 16 hours post treatment with A) 40 µM flg22 peptide or B) *P. infestans* culture filtrate (CF). C) Relative expression, compared to untreated plants (0), of known PTI marker genes *NbPti5*, *NbAcre31*, *NbWRKY7* and *NbWRKY8* at 16, 24 and 48 hours post infection with WT 88069 *P. infestans*. Error bars are standard error.
(PDF)

Figure S10 ***NbNTP1*** **and** ***NbNTP2*** **transcripts accumulate following treatment with** ***P. infestans*** **culture filtrate but not flg22.** Graph shows relative expression, compared to untreated plants (0) of *NbNTP1* and *NbNTP2* at 3 and 16 hours post treatment with either *P. infestans* culture filtrate (CF) or 40 µM flg22. Error bars are standard error.
(PDF)

Figure S11 **Treatment with** ***P. infestans*** **culture filtrate reduces GFP-StNTP1 and GFP-StNTP2 fluorescence.** Confocal images showing representative levels of GFP-StNTP1 and GFP-StNTP2 fluorescence in the presence and absence of *P. infestans* culture filtrate (CF). Scale bar is 50 µm.
(PDF)

Figure S12 ***P. infestans*** **culture filtrate but not flg22 triggers NTP re-localisation from the ER to the nucleus.** A: Confocal images showing representative nuclei indicating presence or absence of GFP-StNTP1 and GFP-StNTP2 nuclear fluorescence with each of the treatments shown, scale bar is 5 µm. B: Graph shows the percentage of nuclei containing either GFP-StNTP1 or GFP-StNTP2 fluorescence with each treatment. The numbers of nuclei showing fluorescence out of the number examined is shown above each bar on the graph.
(PDF)

Figure S13 **Nuclear accumulation of GFP-StNTP1ΔTM and GFP-StNTP22ΔTM fusion proteins is unaffected by Pi03192.** Confocal images show A. GFP-StNTP1ΔTM and pFlub empty vector, B. GFP-StNTP1ΔTM and pFlub-03192, C. GFP-StNTP2ΔTM and pFlub empty vector and D. GFP-StNTP2ΔTM and pFlub-03192. Scale bar is 50 µm.
(PDF)

Table S1 **Primer sequences.** Sequences of primers used in cloning and for qRT-PCR analysis.
(XLSX)
