# Supplementary figures and images for: An RxLR Effector from Phytophthora infestans Prevents Re-localisation of Two Plant NAC Transcription Factors from the Endoplasmic Reticulum to the Nucleus
Source: PLoS Pathog. 2013 Oct 10;9(10):e1003670. doi: 10.1371/journal.ppat.1003670 (PMC3795001; doi:10.1371/journal.ppat.1003670)

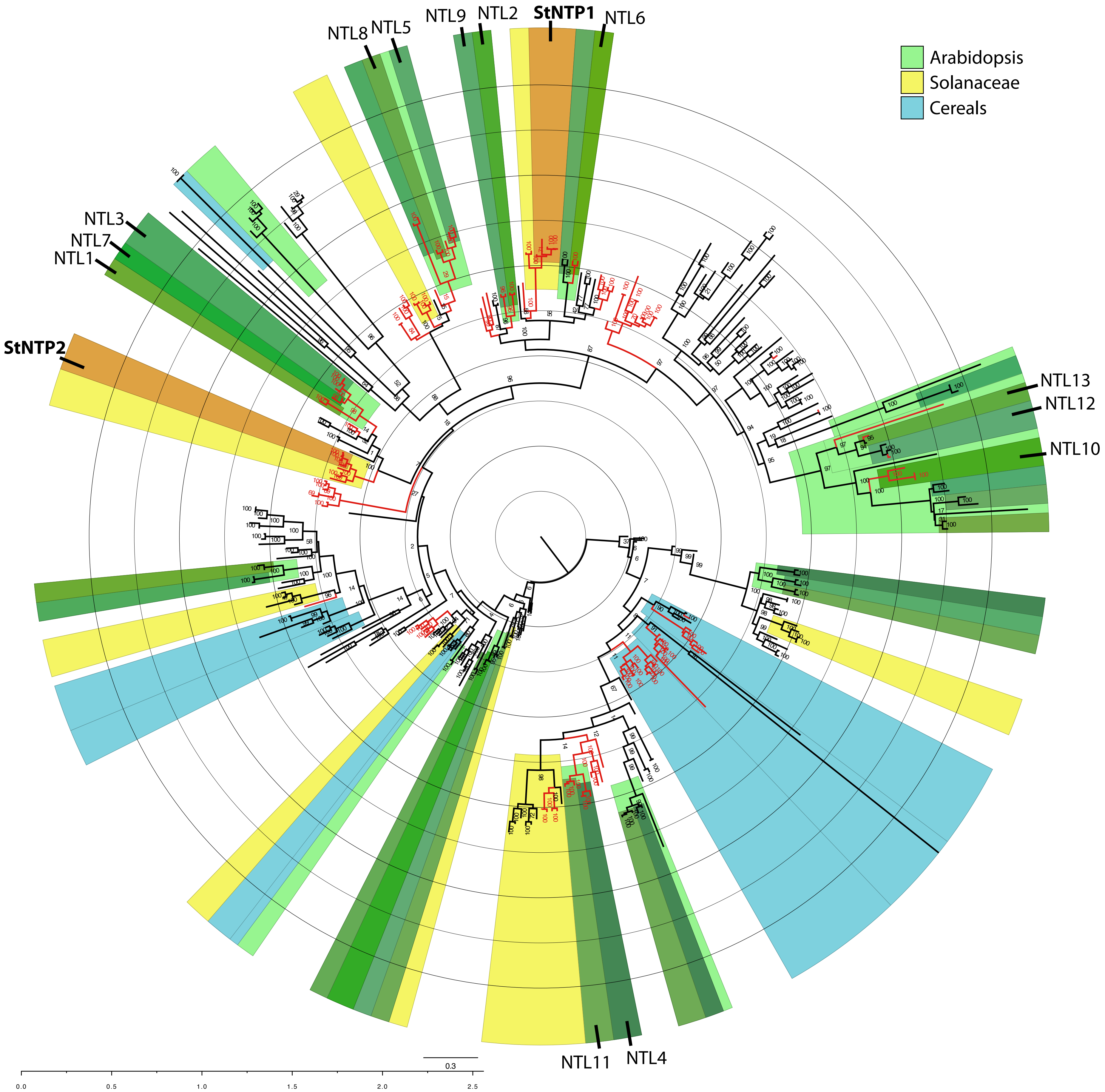

Supplement: Figure S1 — Phylogenetic tree of plant NAC transcription factors. Maximum Likelihood phylogenetic reconstruction for 337 NAM domains, extracted from plant NAC transcription factors. Red branches indicate NAC TFs that also possess a predicted C-terminal transmembrane domain. Clades are coloured by group for their originating plants: Arabidopsis (green; several shades are used for visual separation of clades), Solanaceae (yellow; orange used to indicate clades containing StNTP1 and StNTP2) and cereals (blue). Sequences are divided into 40 clades that each have 100% bootstrap support at the distal node. Of the previously classified AtNTL proteins, StNTP1 is most closely associated with AtNTL6, and StNTP2 equally with AtNTL1, AtNTL7 and AtNTL3. However, there is no clear one-to-one association of any StNTP with any AtNTL. Notable features include: the presence of many homogeneous TM domain-containing clades; the absence of grass sequences in the clades containing NTL proteins; and the apparent expansion of NAM domain-containing proteins in Arabidopsis. (PDF) [file ppat.1003670.s001.pdf]

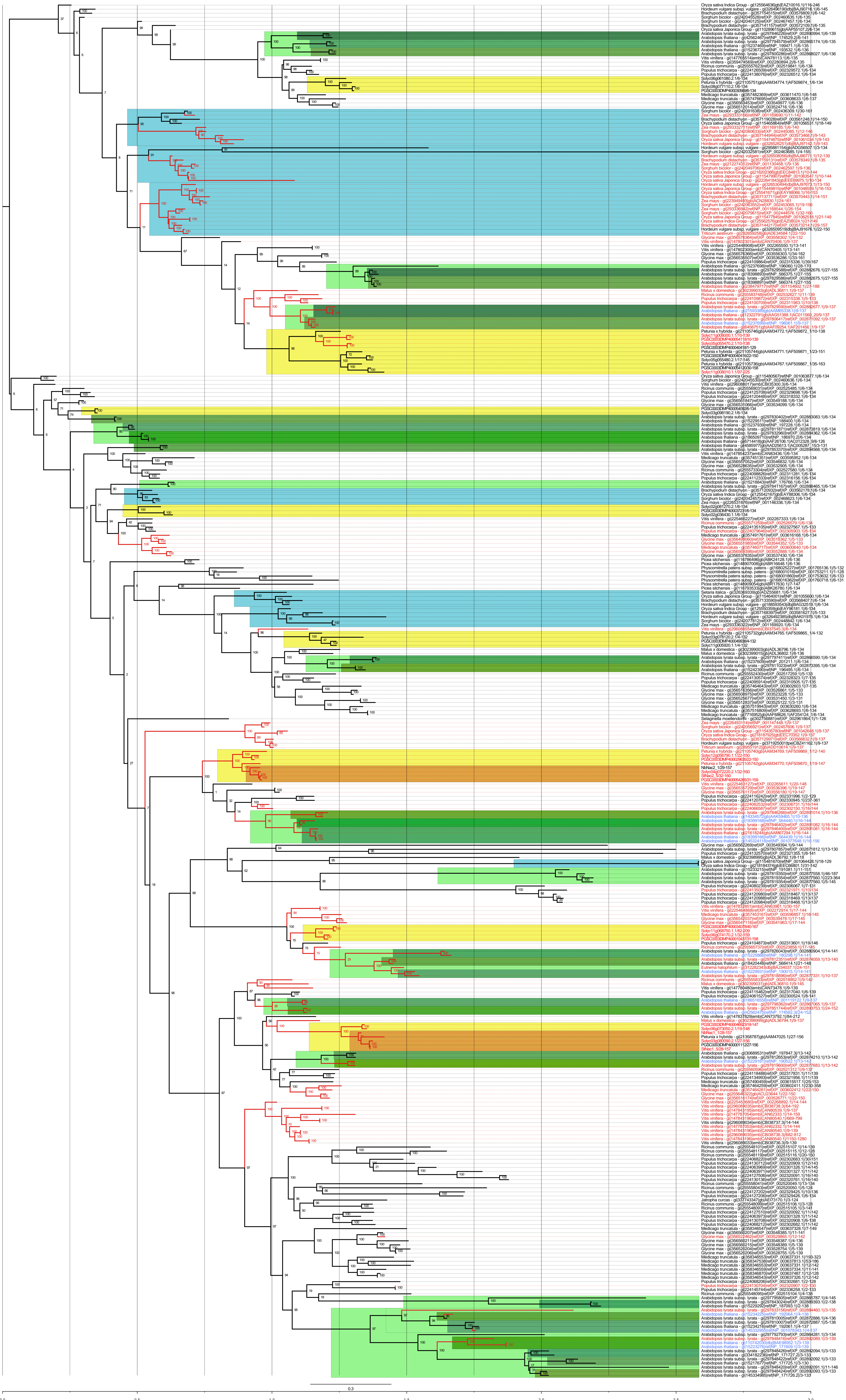

Supplement: Figure S2 — Phylogenetic tree of plant NAC transcription factors. Maximum Likelihood phylogenetic reconstruction for 337 NAM domains, extracted from plant NAC transcription factors. This figure is identical to Figure S1, except for layout and the individual labelling of proteins. Proteins labelled in blue are Arabidopsis AtNTL proteins, and those labelled in red are predicted to contain a C-terminal transmembrane domain. (PDF) [file ppat.1003670.s002.pdf]

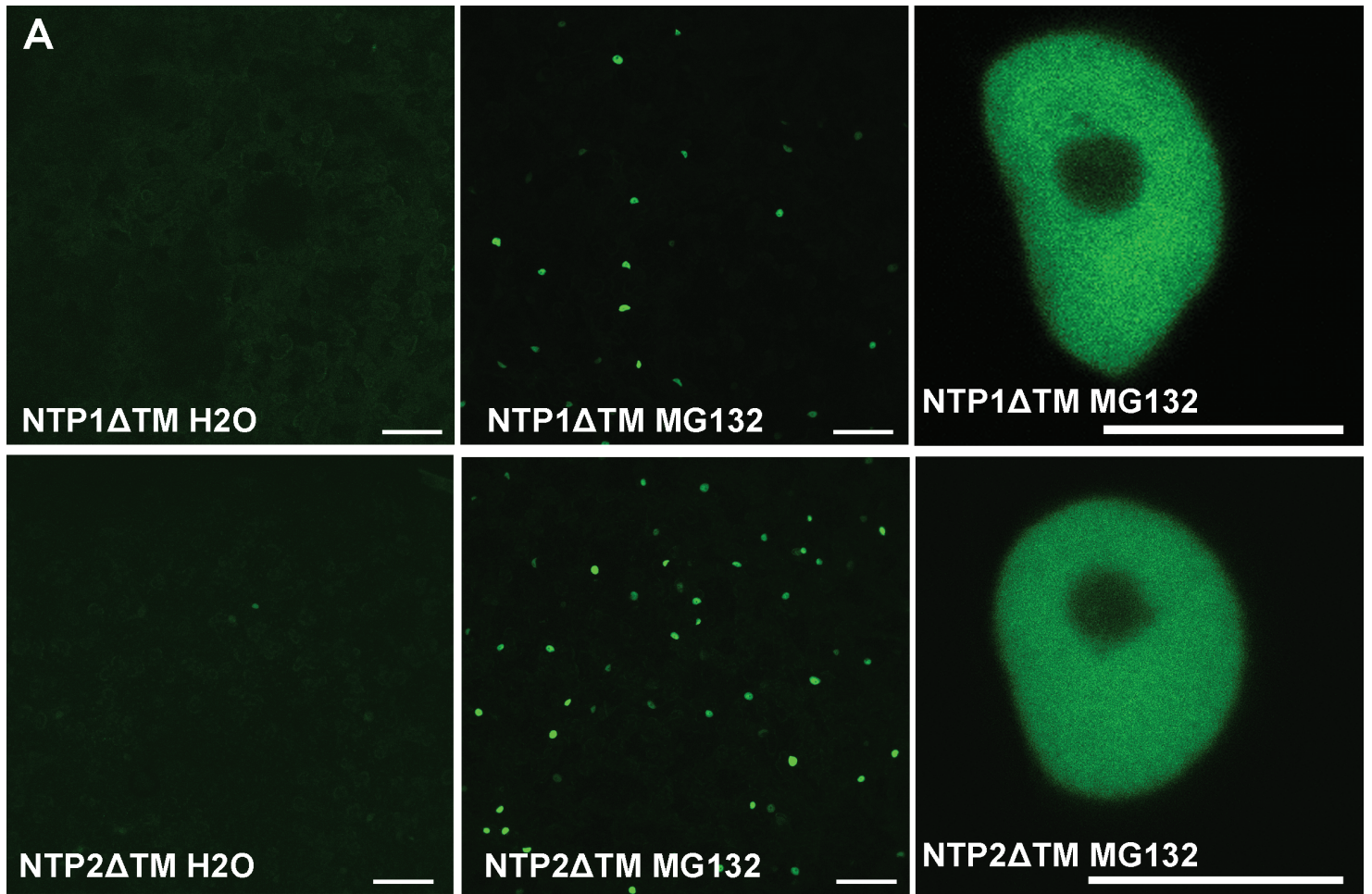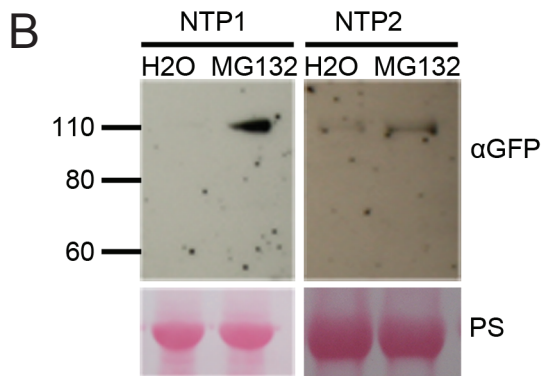

Supplement: Figure S4 — Localisation and stability of delta TM NAC constructs. A. Confocal images of GFP-StNTP1ΔTM and GFP-StNTP2ΔTM plus or minus MG132 treatment. The first two panels of each row are images with a x20 lens with scale bars representing 100 µm. The last panel of each row are images using a x64 lens zoomed in on a single nucleus with the scale bars representing 10 µm. B. Immunoblots of GFP-StNTP1ΔTM and GFP-StNTP2ΔTM plus or minus MG132 treatment probed with a specific GFP antibody, numbered ladder on the left represent size in kDa and PS is ponceau staining. (PDF) [file ppat.1003670.s004.pdf]

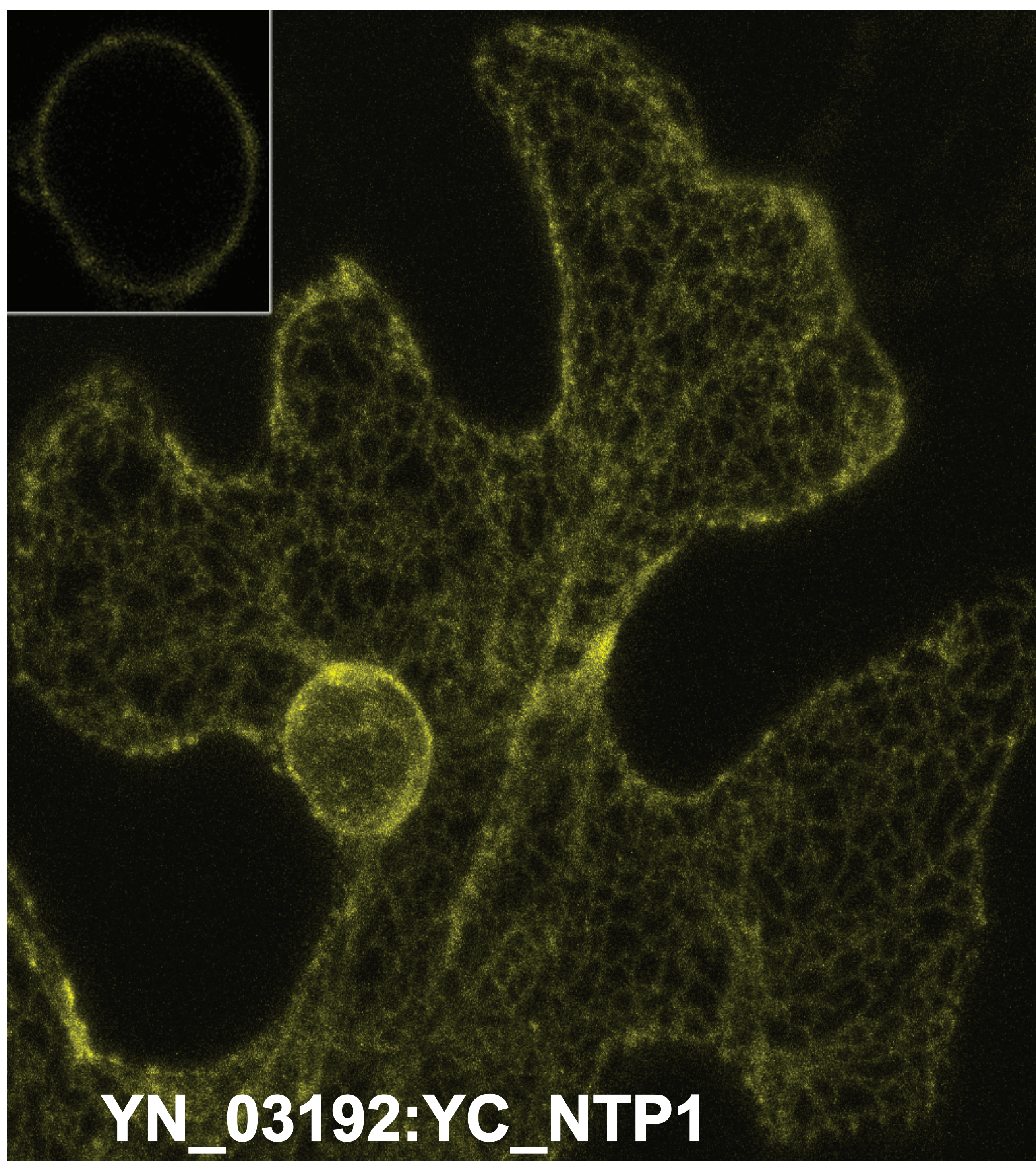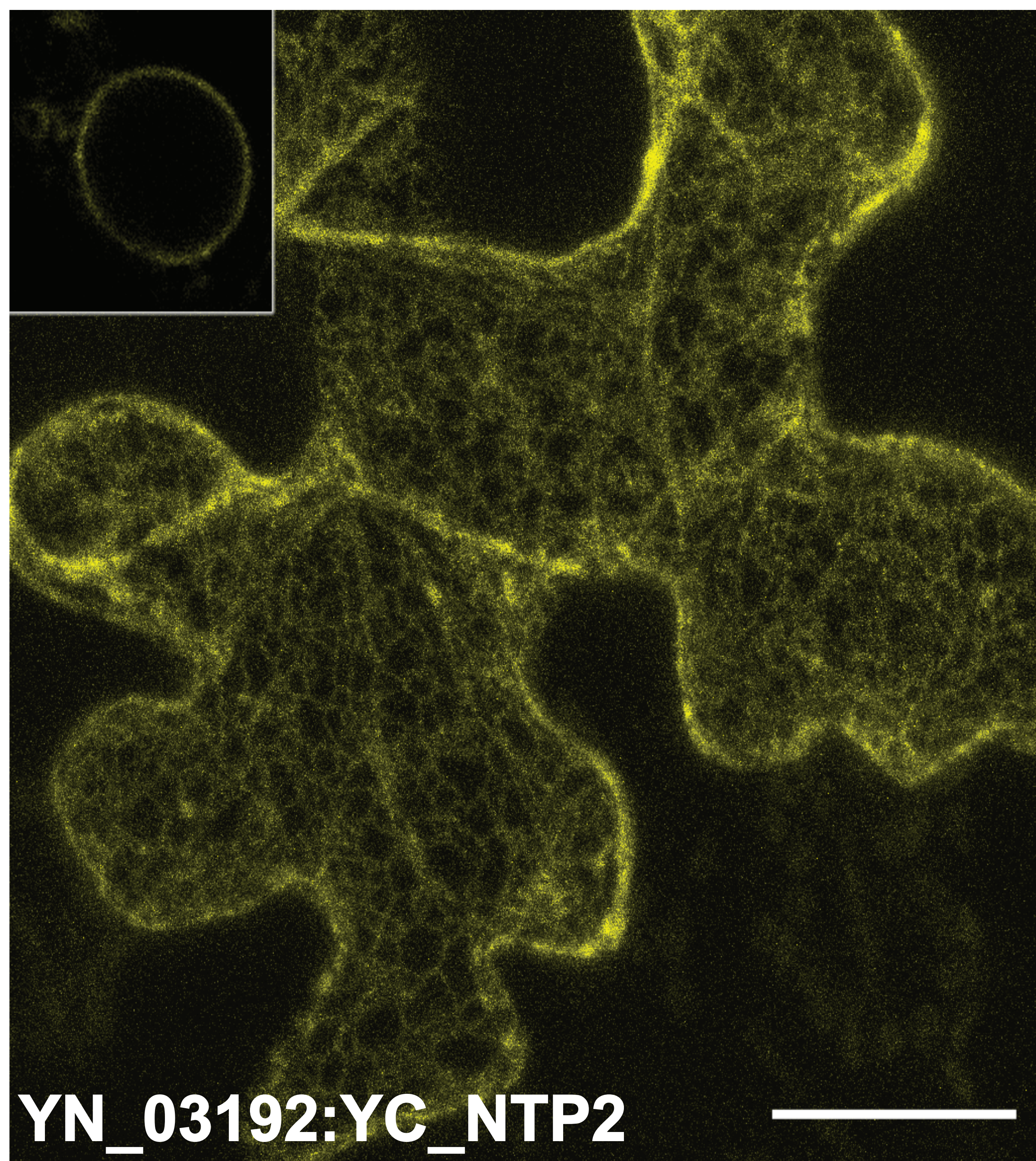

Supplement: Figure S5 — Split YFP of ER localised interaction of Pi03192 and StNTPs. Confocal images of YC-StNTP1 or YC-StNTP2 co-expressed with YN-Pi03192 showing clear ER localisation with inset slices showing the ER around the nucleus. Scale bars are 10 µm. (PDF) [file ppat.1003670.s005.pdf]

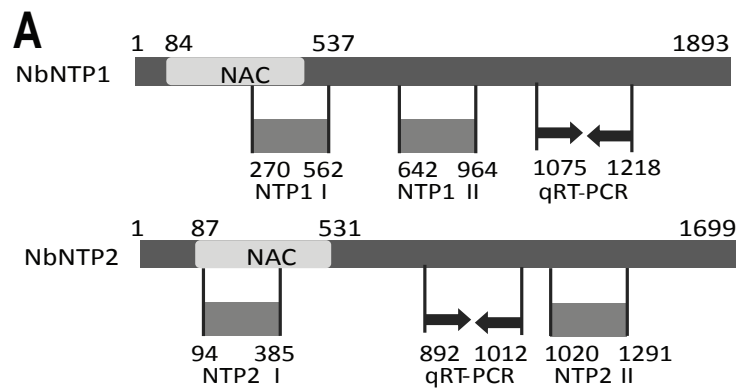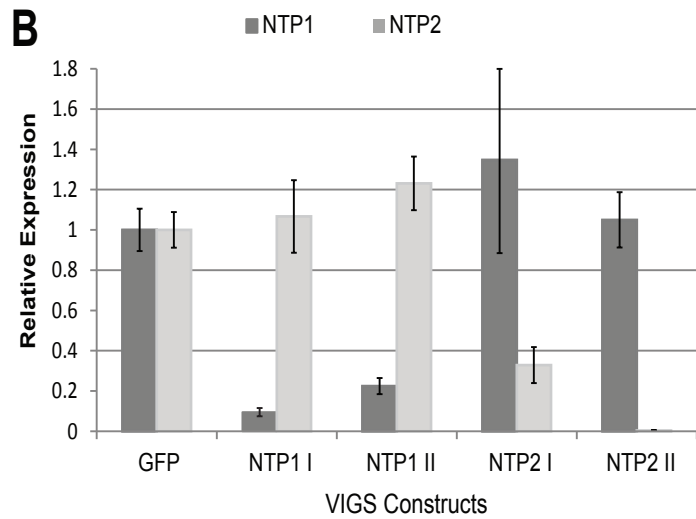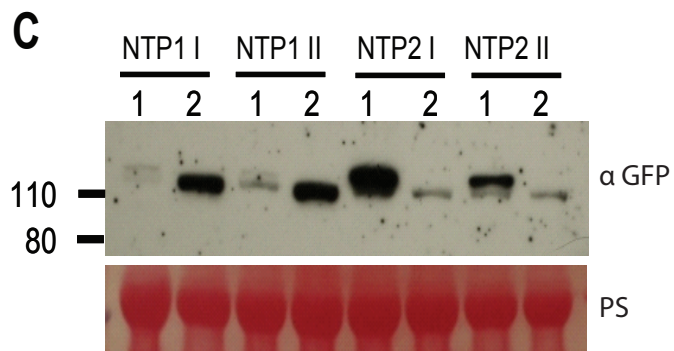

Supplement: Figure S6 — NTP VIGS constructs and gene transcript and protein levels. A. Schematic representations of the NbNTP1 and NbNTP2 genes showing the location of the region used to make each VIGS construct and the location of the qRT-PCR primers (arrows). B. Graph shows relative expression of the NbNTP1 and NbNTP2 genes in each VIGS line with the unsilenced (GFP) control set to 1. Error bars are standard error. C. Immunoblot showing the accumulation of GFP-StNTP1 and GFP-StNTP2 in unsilenced plants (GFP) and plants expressing each of the VIGS constructs as indicated, probed with a specific GFP antibody. PS is Ponceau stain. Sizes are indicated in kD. (PDF) [file ppat.1003670.s006.pdf]

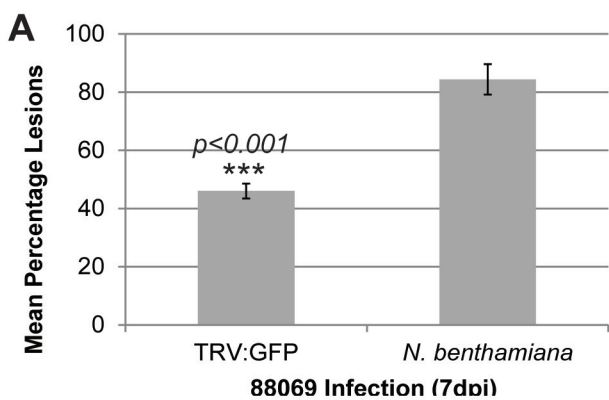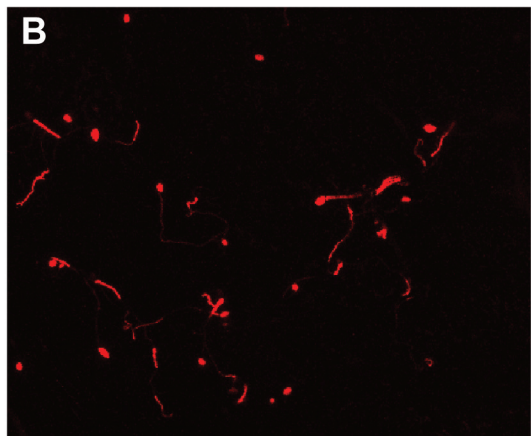

Surface Growth

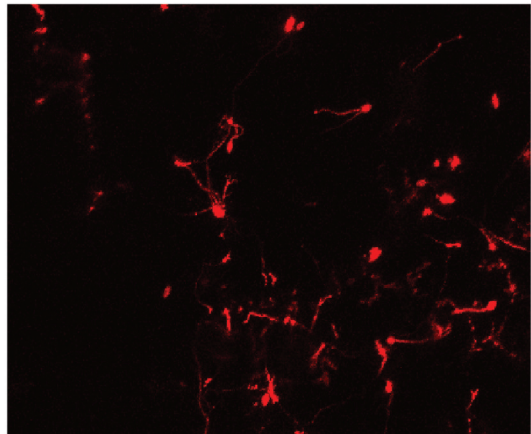

Initial Colonisation

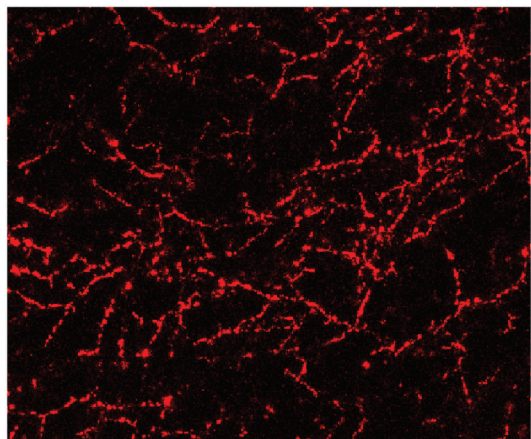

Extensive Mycelial Growth

Supplement: Figure S7 — Early infection categories for P. infestans 88069-tdT. A: Graph shows the presence of TRV significantly reduces the rate of P. infestans colonisation compared to non TRV plants (two tailed t-test p<0.001, n = 6), error bars are standard error. B: Representative confocal images showing what each infection category looks like, as measured in Figure 5B. Surface growth shows sporangia, germinating sporangia and infection hyphae on the leaf surface. Initial colonisation shows the above but with the addition of some hyphal penetration of the tissue immediately below the site of inoculation. Extensive mycelial growth shows mycelial growth of hyphae through the leaf mesophyll layers extending beyond the site of inoculation. (PDF) [file ppat.1003670.s007.pdf]

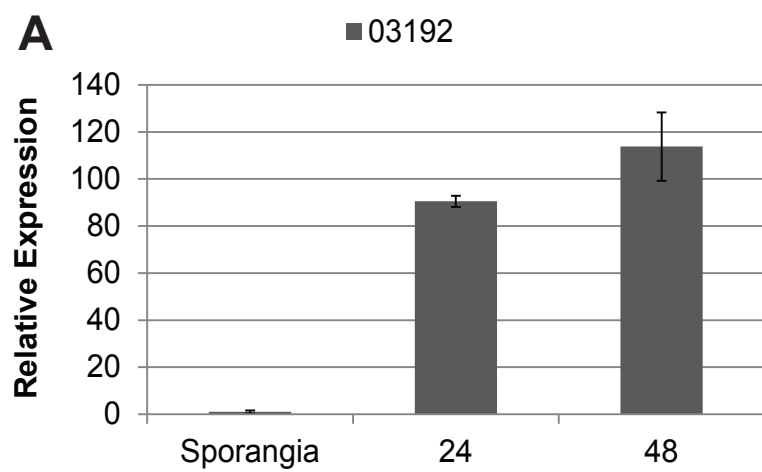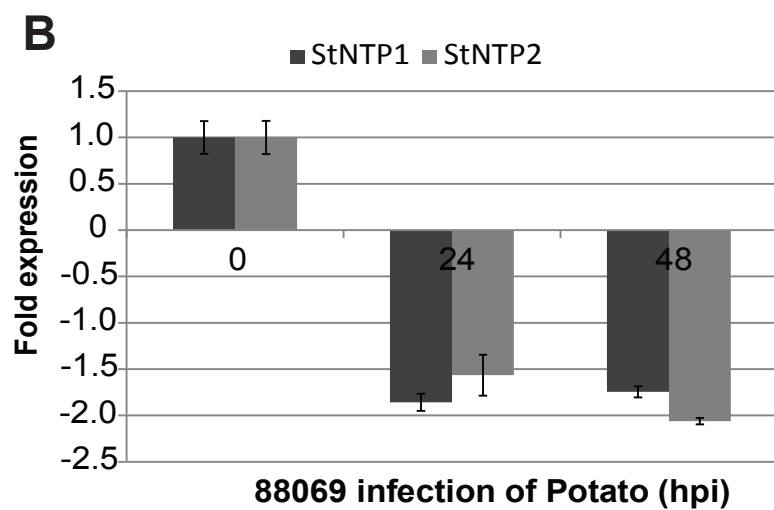

Supplement: Figure S8 — QRT-PCR of P. infestans /potato infection. Expression of A. Pi03192 and B. StNTP1 and StNTP2 at 24 and 48 hours post-inoculation of potato cv Bintje plants infected with wildtype P. infestans. Error bars are standard error. (PDF) [file ppat.1003670.s008.pdf]

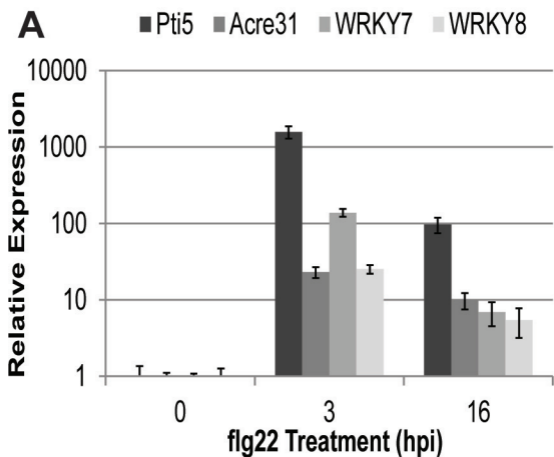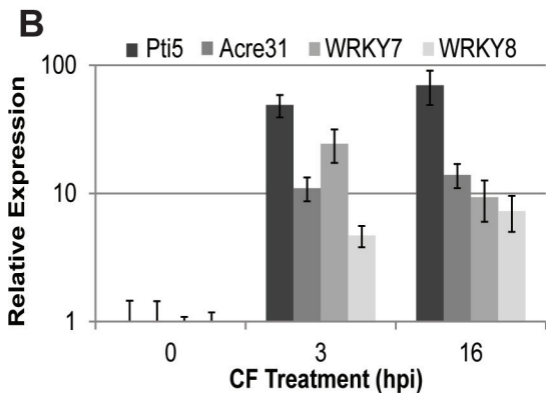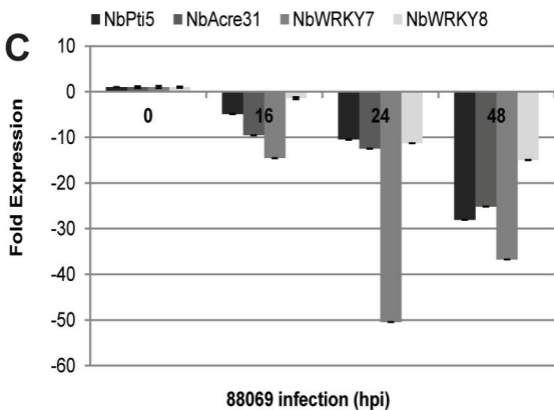

Supplement: Figure S9 — QRT-PCR of PTI marker genes following flg22 and culture filtrate treatments and during infection. Relative expression, compared to untreated plants (0), of known PTI marker genes NbPti5, NbAcre31, NbWRKY7 and NbWRKY8 at 3 and 16 hours post treatment with A) 40 µM flg22 peptide or B) P. infestans culture filtrate (CF). C) Relative expression, compared to untreated plants (0), of known PTI marker genes NbPti5, NbAcre31, NbWRKY7 and NbWRKY8 at 16, 24 and 48 hours post infection with WT 88069 P. infestans. Error bars are standard error. (PDF) [file ppat.1003670.s009.pdf]

■ NbNTP1 flg22 ■ NbNTP1 CF ■ NbNTP2 flg22 ■ NbNTP2 CF

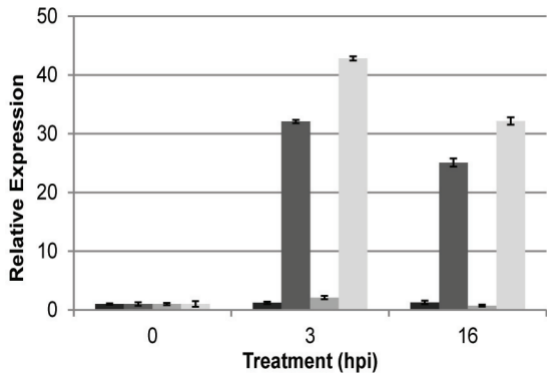

Supplement: Figure S10 — NbNTP1 and NbNTP2 transcripts accumulate following treatment with P. infestans culture filtrate but not flg22. Graph shows relative expression, compared to untreated plants (0) of NbNTP1 and NbNTP2 at 3 and 16 hours post treatment with either P. infestans culture filtrate (CF) or 40 µM flg22. Error bars are standard error. (PDF) [file ppat.1003670.s010.pdf]

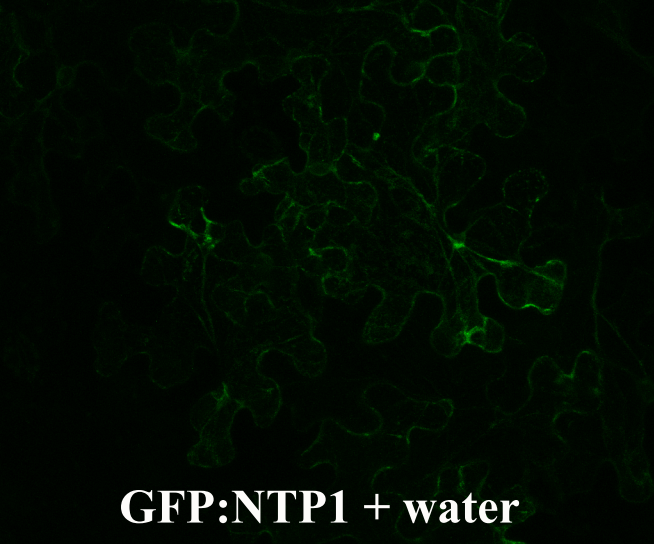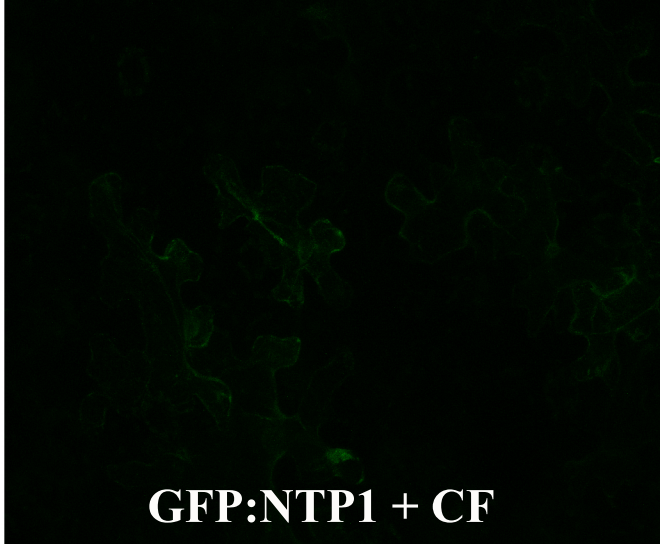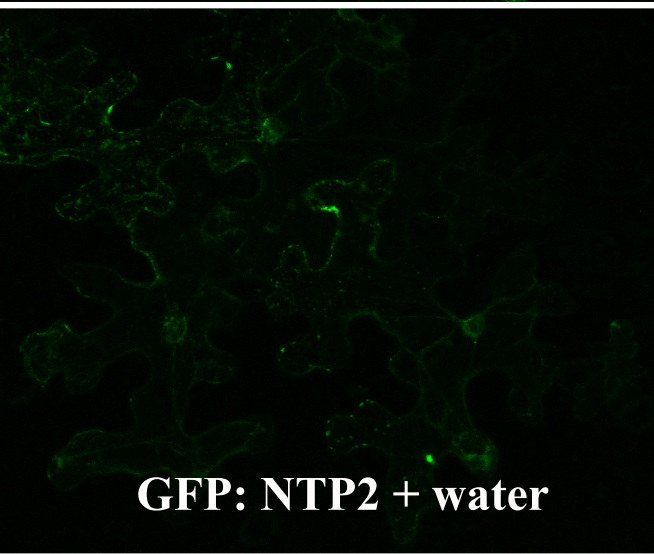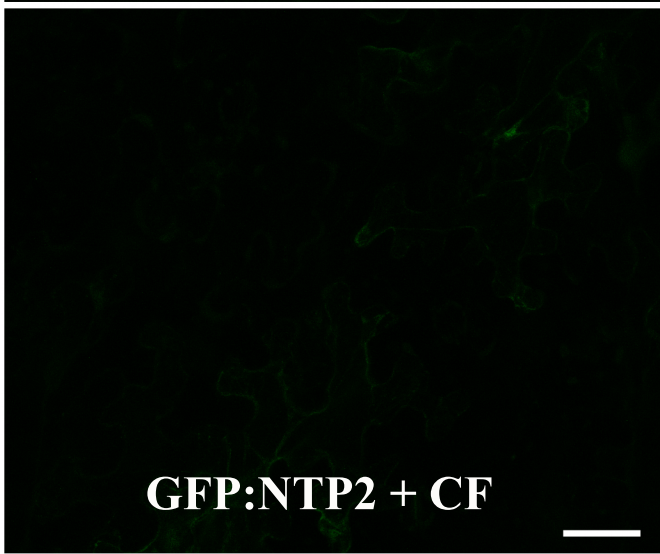

Supplement: Figure S11 — Treatment with P. infestans culture filtrate reduces GFP-StNTP1 and GFP-StNTP2 fluorescence. Confocal images showing representative levels of GFP-StNTP1 and GFP-StNTP2 fluorescence in the presence and absence of P. infestans culture filtrate (CF). Scale bar is 50 µm. (PDF) [file ppat.1003670.s011.pdf]

A

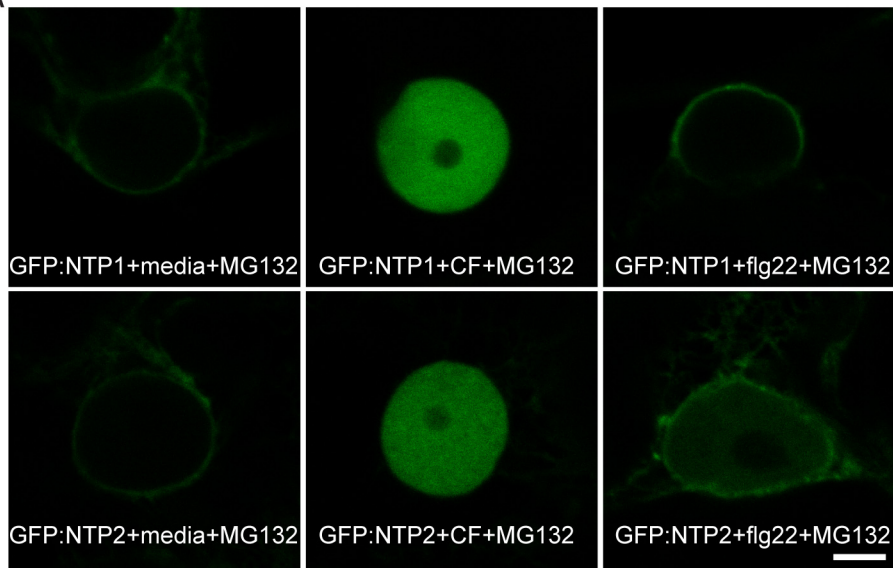

B

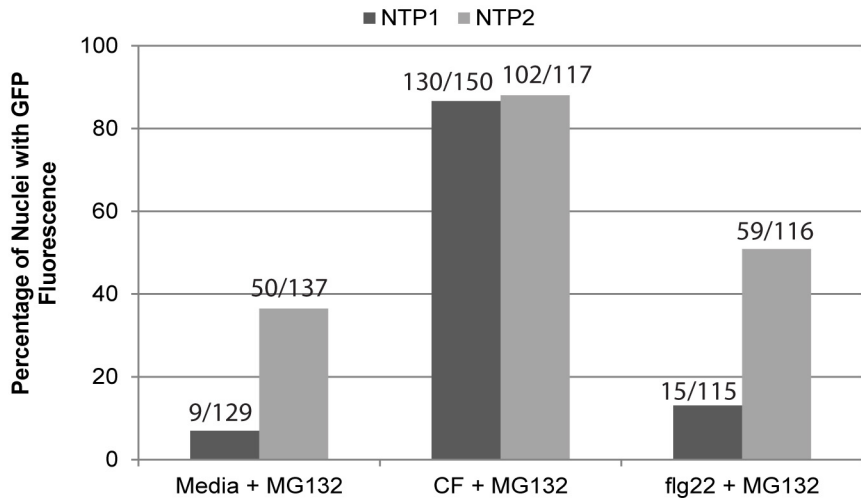

Supplement: Figure S12 — P. infestans culture filtrate but not flg22 triggers NTP re-localisation from the ER to the nucleus. A: Confocal images showing representative nuclei indicating presence or absence of GFP-StNTP1 and GFP-StNTP2 nuclear fluorescence with each of the treatments shown, scale bar is 5 µm. B: Graph shows the percentage of nuclei containing either GFP-StNTP1 or GFP-StNTP2 fluorescence with each treatment. The numbers of nuclei showing fluorescence out of the number examined is shown above each bar on the graph. (PDF) [file ppat.1003670.s012.pdf]

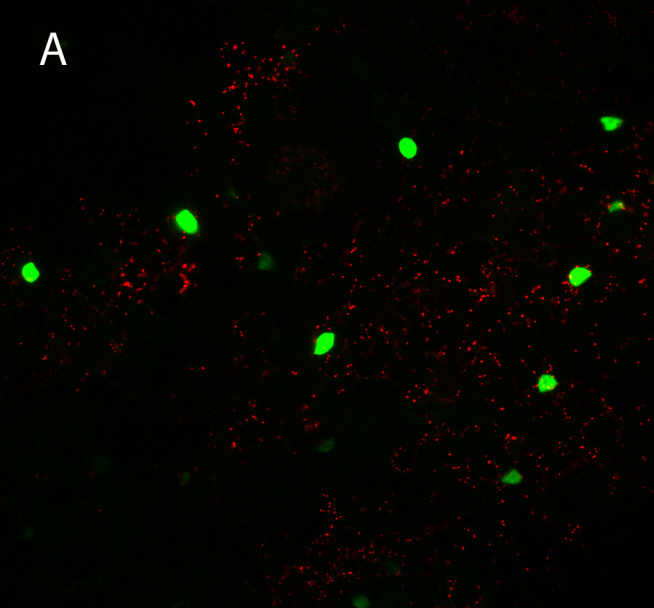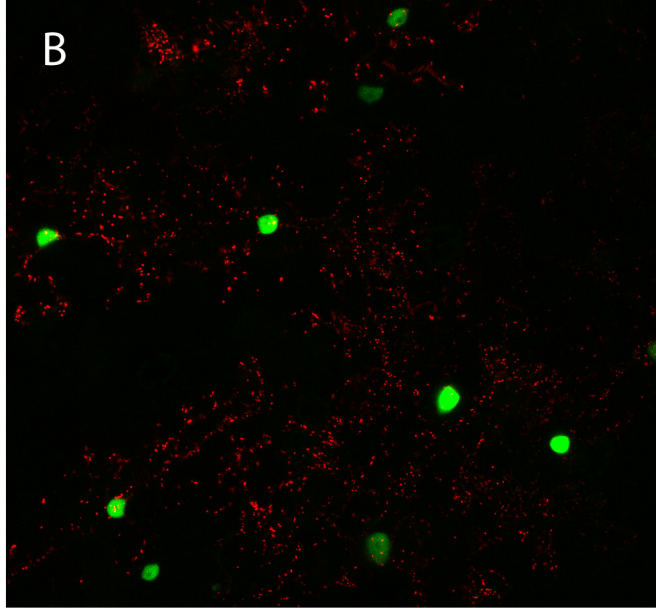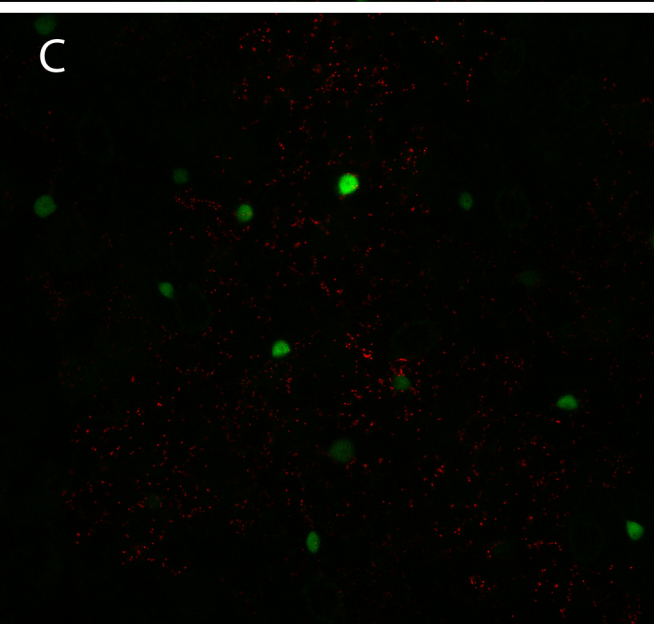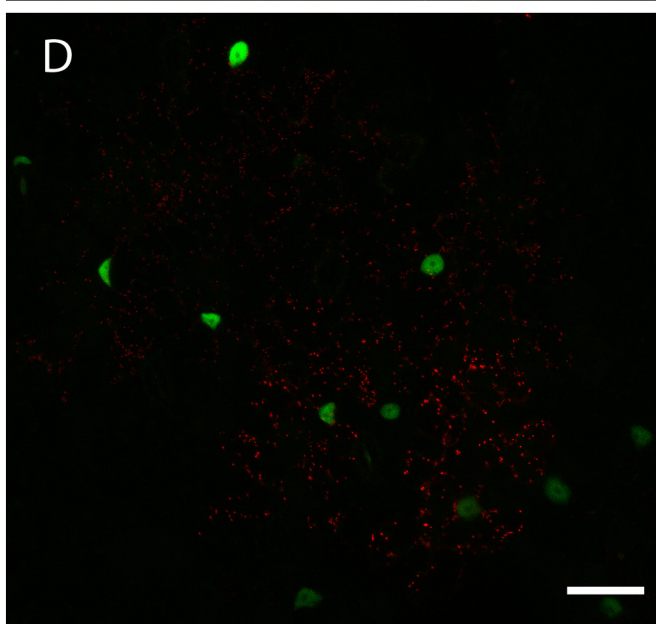

Supplement: Figure S13 — Nuclear accumulation of GFP-StNTP1ΔTM and GFP-StNTP22ΔTM fusion proteins is unaffected by Pi03192. Confocal images show A. GFP-StNTP1ΔTM and pFlub empty vector, B. GFP-StNTP1ΔTM and pFlub-03192, C. GFP-StNTP2ΔTM and pFlub empty vector and D. GFP-StNTP2ΔTM and pFlub-03192. Scale bar is 50 µm. (PDF) [file ppat.1003670.s013.pdf]
