# Supplementary material for: An RxLR Effector from Phytophthora infestans Prevents Re-localisation of Two Plant NAC Transcription Factors from the Endoplasmic Reticulum to the Nucleus
Source: PLoS Pathog. 2013 Oct 10;9(10):e1003670. doi: 10.1371/journal.ppat.1003670 (PMC3795001; doi:10.1371/journal.ppat.1003670)
Supplement: Figure S3 — Alignment of NAC DBDs for StNTPs and AtNTLs. The NAC DNA binding (NAM) domains for the 13 published Arabidopsis NTLs were aligned with the NAM domains of both potato and N. benthamiana NTP1 and NTP2 proteins. Conserved residues are shaded black while those shaded grey share similar properties. Residues essential for DNA binding are marked with a # and those required for NAC dimerization are marked with *. (PDF) [file ppat.1003670.s003.pdf]

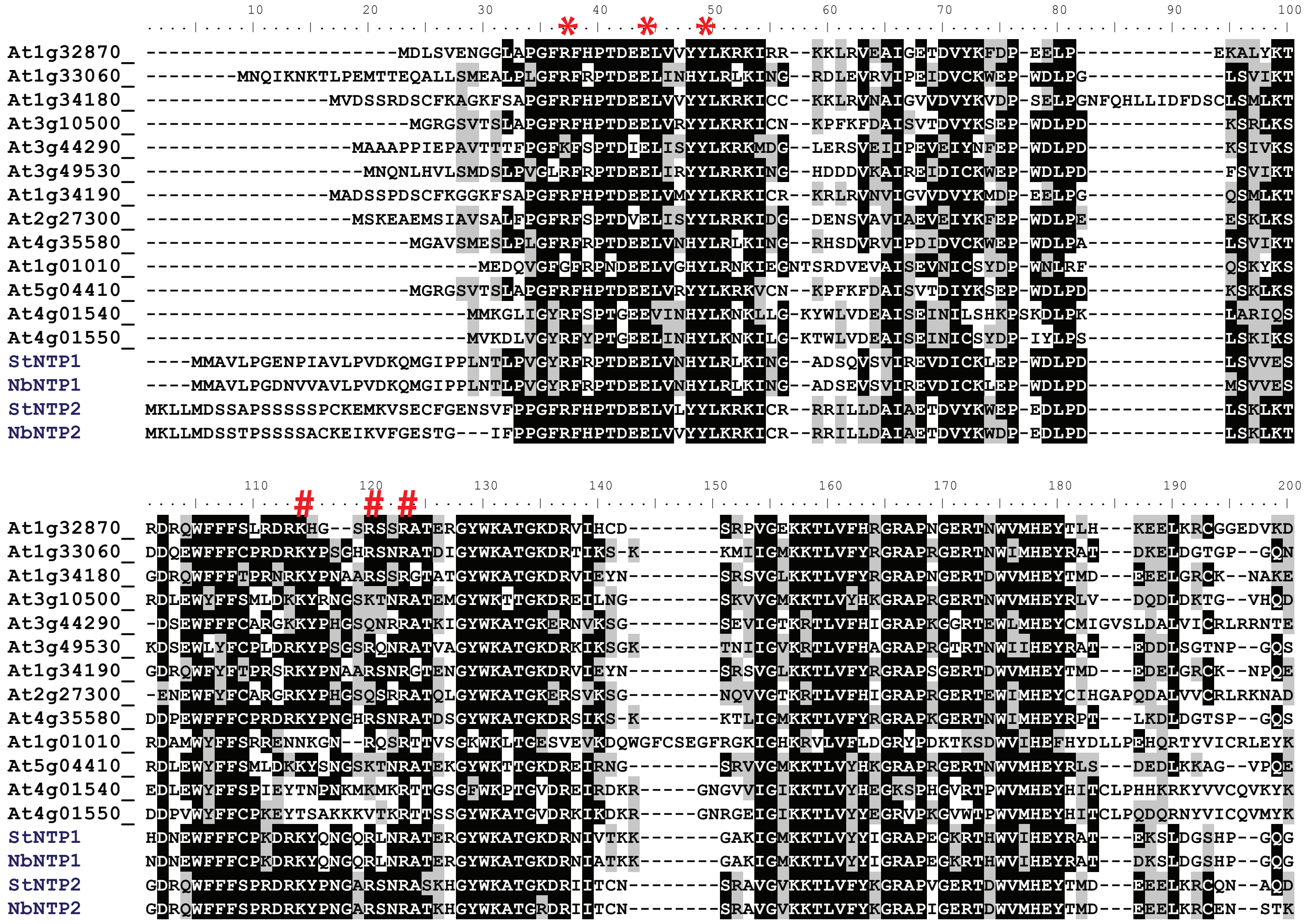
10 20 30 40 50 60 70 80 90 100
At1g32870_ ----------------------MDLSVENGGLAPGFRFHPTDEELVVYYLKRKIRR--KKLRVEAIGETDVYKFDP-EELP------------EKALYKT
At1g33060_ --------MNQIKNKTLPEMTTEQALLSMEALPLGFRFRPTDEELINHYLRLKING--RDLEVRVIPEIDVCKWEP-WDLPG------------LSVIKT
At1g34180_ ----------------MVDSSRDSCFKAGKFSAPGFRFHPTDEELVVYYLKRKICC--KKLRVNAIGVVDVYKVDP-SELPGNFQHLLIDFDSCLSMLKT
At3g10500_ ------------------------MGRGSVTSLAPGFRFHPTDEELVRYYLKRKICN--KPFKFDAISVTDVYKSEP-WDLPD------------KSRLKS
At3g44290_ ------------------MAAAPPIEPAVTTTFPGFKFSPTDIELISYYLKRKMDG--LERSVEIIPEVEIYNFEP-WDLPD------------KSIVKS
At3g49530_ -------------------MNQNLHVLSMDSLPVGLRFRPTDEELIRYYLRRKING--HDDDVKAIREIDICKWEP-WDLPD------------FSVIKT
At1g34190_ ----------------MADSSPDSCFKGGKFSAPGFRFHPTDEELVMYYLKRKICR--KRLRVNVIGVVDVYKMDP-EELPG------------QSMLKT
At2g27300_ ------------------MSKEAEMSIAVSALFPGFRFSPTDVELISYYLRRKIDG--DENSVAVIAEVEIYKFEP-WDLPE------------ESKLKS
At4g35580_ ------------------------MGAVSMESLPLGFRFRPTDEELVNHYLRLKING--RHSDVRVIPDIDVCKWEP-WDLPA------------LSVIKT
At1g01010_ -----------------------------MEDQVGFGFRPNDEELVGHYLRNKIEGNTSRDVEVAISEVNICSYDP-WNLRF------------QSKYKS
At5g04410_ ------------------------MGRGSVTSLAPGFRFHPTDEELVRYYLKRKVCN--KPFKFDAISVTDIYKSEP-WDLPD------------KSKLKS
At4g01540_ ---------------------------MMKGLIGYRFSPTGEEVINHYLKNKLLG-KYWLVDEAISEINILSHKPSKDLPK------------LARIQS
At4g01550_ ---------------------------MVKDLVGYRFYPTGEELINHYLKNKILG-KTWLVDEAISEINICSYDP-IYLPS------------LSKIKS
StNTP1 ----MMAVLPGENPIAVLPVDKQMGIPPLNTLPVGYRFRPTDEELVNHYLRLKING--ADSQVSVIREVDICKLEP-WDLPD------------LSVVES
NbNTP1 ----MMAVLPGDNVVAVLPVDKQMGIPPLNTLPVGYRFRPTDEELVNHYLRLKING--ADSEVSVIREVDICKLEP-WDLPD------------MSVVES
StNTP2 MKLLMDSSAPSSSSSPCKEMKVSECFGENSVFPPGFRFHPTDEELVLYYLKRKICR--RRILLDAIAETDVYKWEP-EDLPD------------LSKLKT
NbNTP2 MKLLMDSSTPSSSSACKEIKVFGESTG---IFPPGFRFHPTDEELVVYYLKRKICR--RRILLDAIAETDVYKWDP-EDLPD------------LSKLKT
110 120 130 140 150 160 170 180 190 200
At1g32870_ RDRQWFFFSLRDRKHG--SRSSRATERGYWKATGKDRVIHCD--------SRPVGEKKTLVFHRGRAPNGERTNWVMHEYTLH---KEELKRCGGEDVKD
At1g33060_ DDQEWFFFCPRDRKYPSGHRSNRATDIGYWKATGKDRTIKS-K--------KMIIGMKKTLVFYRGRAPRGERTNWIMHEYRAT---DKELDGTGP--GQN
At1g34180_ GDRQWFFFTPRNRKYPNAARSSRGTATGYWKATGKDRVIEYN--------SRSVGLKKTLVFYRGRAPNGERTDWVMHEYTMD---EEELGRCK--NAKE
At3g10500_ RDLEWYFFSMLDKKYRNGSKTNRATEMGYWKTTGKDREILNG--------SKVVGMKKTLVYHKGRAPRGERTNWVMHEYRLV---DQDLDKTG--VHQD
At3g44290_ -DSEWFFFCARGKKYPHGSQNRRATKIGYWKATGKERNVKSG--------SEVIGTKRTLVFHIGRAPKGGRTEWLMHEYCMIGVSLDALVICRLRRNTE
At3g49530_ KDSEWLYFCPLDRKYPSGSRQNRATVAGYWKATGKDRKIKSGK-------TNIIGVKRTLVFHAGRAPRGTRTNWIIHEYRAT---EDDLSGTNP--GQS
At1g34190_ GDRQWFYFTPRSRKYPNAARSNRGTENGYWKATGKDRVIEYN--------SRSVGLKKTLVFYRGRAPSGERTDWVMHEYTMD---EDELGRCK--NPQE
At2g27300_ -ENEWFYFCARGRKYPHGSQSRRATQLGYWKATGKERSVKSG--------NQVVGTKRTLVFHIGRAPRGERTEWIMHEYCIHGAPQDALVVCRLRKNAD
At4g35580_ DDPEWFFFCPRDRKYPNGHRSNRATDSGYWKATGKDRSIKS-K--------KTLIGMKKTLVFYRGRAPKGERTNWIMHEYRPT---LKDLDGTSP--GQS
At1g01010_ RDAMWYFFSRRENNKGN--RQSRTTVSGKWKLTGESVEVKDQWGFCSEGFRGKIGHKRVLVFLDGRYPDKTKSDWVIHEFHYDLLPEHQRTYVICRLEYK
At5g04410_ RDLEWYFFSMLDKKYSNGSKTNRATEKGYWKTTGKDREIRNG--------SRVVGMKKTLVYHKGRAPRGERTNWVMHEYRLS---DEDLKKAG--VPQE
At4g01540_ EDLEWYFFSPIEYTNPNKMKMKRTTGSGFWKPTGVDREIRDKR-----GNGVVIGIKKTLVYHEGKSPHGVRTPWVMHEYHITCLPHHKRKYVVCQVKYK
At4g01550_ DDPVWYFFCPKEYTSAKKKVTKRTTSSGYWKATGVDRKIKDKR-----GNRGEIGIKKTLVYYEGRVPKGVWTPWVMHEYHITCLPQDQRNYVICQVMYK
StNTP1 HDNEWFFFCPKDRKYQNGQRLNRATERGYWKATGKDRNIVTKK-------GAKIGMKKTLVYYIGRAPEGKRTHWVIHEYRAT---EKSLDGSHP--GQG
NbNTP1 NDNEWFFFCPKDRKYQNGQRLNRATERGYWKATGKDRNIATKK-------GAKIGMKKTLVYYIGRAPEGKRTHWVIHEYRAT---DKSLDGSHP--GQG
StNTP2 GDRQWFFFSPRDRKYPNGARSNRASKHGYWKATGKDRIITCN--------SRAVGVKKTLVFYKGRAPVGERTDWVMHEYTMD---EEELKRCQN--AQD
NbNTP2 GDRQWFFFSPRDRKYPNGARSNRATKHGYWKATGRDRIITCN--------SRAVGVKKTLVFYKGRAPIGERTDWVMHEYTMD---EEELKRCEN--STK
